# Combinatorial epigenetic patterns as quantitative predictors of chromatin biology

**DOI:** 10.1186/1471-2164-15-76

**Published:** 2014-01-28

**Authors:** Marcin Cieślik, Stefan Bekiranov

**Affiliations:** 1 Department of Biochemistry and Molecular Genetics, University of Virginia Health System, Charlottesville, Virginia, USA

## Abstract

**Background:**

Chromatin immunoprecipitation followed by deep sequencing (ChIP-seq) is the most widely used method for characterizing the epigenetic states of chromatin on a genomic scale. With the recent availability of large genome-wide data sets, often comprising several epigenetic marks, novel approaches are required to explore functionally relevant interactions between histone modifications. Computational discovery of "chromatin states" defined by such combinatorial interactions enabled descriptive annotations of genomes, but more quantitative approaches are needed to progress towards predictive models.

**Results:**

We propose non-negative matrix factorization (NMF) as a new unsupervised method to discover combinatorial patterns of epigenetic marks that frequently co-occur in subsets of genomic regions. We show that this small set of combinatorial "codes" can be effectively displayed and interpreted. NMF codes enable dimensionality reduction and have desirable statistical properties for regression and classification tasks. We demonstrate the utility of codes in the quantitative prediction of Pol2-binding and the discrimination between Pol2-bound promoters and enhancers. Finally, we show that specific codes can be linked to molecular pathways and targets of pluripotency genes during differentiation.

**Conclusions:**

We have introduced and evaluated a new computational approach to represent combinatorial patterns of epigenetic marks as quantitative variables suitable for predictive modeling and supervised machine learning. To foster widespread adoption of this method we make it available as an open-source software-package – epicode at https://github.com/mcieslik-mctp/epicode.

## Background

Biochemical and structural properties of chromatin are implicated in the function and maintenance of genomes (*e.g.*[1]). Chromatin immunoprecipitation followed by deep sequencing (ChIP-seq) is becoming the standard method for the genome-wide mapping of histone modifications and transcription factor (TF) binding sites [2].

The analysis and interpretation of ChIP-seq data sets is a difficult task [3]. Most of the existing analysis tools are focused on the delineation of enriched sites from a single sample with optional "input control" [4]. For histone modifications this task becomes more challenging as their enrichments are often weaker and less localized. A number of groups have extended the peak-calling approach to identify broad domains [5,6] or analytically represent ChIP-signals beyond read-counts [7]. In order to link epigenetic marks to biological functions and processes, peak calling has also been adapted to paired experimental designs [8]. Individually, each epigenetic mark provides some data towards understanding the structure and biochemistry of the underlying genome. However, it has been argued that the cooperative action of multiple histone modifications, variants, and TFs is functionally most informative [9,10]. Unfortunately, none of the standard peak-based method deals with multiple marks and the reconciliation of several sets of peaks is an added challenge [11-13].

An alternative, and orthogonal, approach is to integrate individual histone modification maps to discover latent relationships between epigenetic marks. Broadly, these approaches fall into two categories: genome-wide segmentation and locus-based clustering. For example, ChromHMM and Segway [14,15] partition the genome into epigenetically-similar regions and have been able to reliably associate chromatin profiles with transcription start sites and putative enhancer regions [16]. Similarly, clustering approaches, such as ChromaSig, attempt to identify loci with globally congruent "chromatin signatures". Although the two types of methods differ greatly in the statistical modeling of data, they make the general assumption that a small set of "chromatin states" is sufficient to annotate the genome [17]. Experimental results suggests that these models are too restricted to capture the genome-wide variability of chromatin patterns [16]. The number of global "chromatin states" has been estimated to be in the several hundreds even when only a small set of marks is used to define each pattern [18].

Both clustering and segmentation results in the hard assignment of a single "chromatin signature" to each locus. This allows for certain types of functional enrichment analyses [19], but is not, in general, conductive to quantitatively link "chromatin state" to genome biology. Regression and other supervised machine learning technique are needed to move from descriptive annotations to quantitative and predictive models [20]. In most of these approaches, levels of epigenetic signals are linked to a biologically important readout (*e.g.* transcript level [21,22] or polymerase occupancy [20]). Unfortunately, histone modifications tend to be highly correlated, which makes it difficult to asses the relative importance of the variables (marks) [23]. Since these problems are further exacerbated during stepwise regression, it is difficult to explain how, in terms of direction and strength, combinatorial interactions between marks are linked to the biological readout [24].

Here, we describe a novel method based on non-negative matrix factorization (NMF) to discover combinatorial patterns of epigenetic marks from integrated epigenetic data sets. Locus-specific weights of these mark co-occurrence patterns are used as quantitative variables, suitable for regression and supervised machine learning. We are able to demonstrate that basis patterns are quantitative predictors of biochemical activity, discriminate between classes of genomic regions, and are associated with molecular pathways. Hence we propose to call these patterns *bona fide* epigenetic "codes". In the remaining sections we describe the basic algorithm and its extensions (Formulation), investigate important statistical properties of basis patterns (Properties), and show their utility in regression, classification, and gene set analysis (Case Studies). A reference implementation of the method is available at https://github.com/mcieslik-mctp/epicode and in (Additional file 1).

## Results

### Formulation

The total number of distinct "chromatin states" in the genome is likely inestimable, but clearly specific combinations of a small number of marks are associated with distinct functions or region classes [18,25]. Rather than trying to delineate global "chromatin states", we attempt to identify patterns of marks that frequently co-occur in subsets of genomic regions. We anticipate marks within a combinatorial pattern to be "written" or "erased" by the same chromatin remodeling complex or during the same reprogramming event, which results in their high correlation. Along the lines of the original "histone code" hypothesis [9] we expect these patterns to either, encode biochemical signals that are recognized by multivalent epigenetic "readers" [26], or to represent coordinated epigenetic regulation [27,28]. We introduce a method which represents the full set of histone modifications or variants occurring at a selected annotation class (*i.e.*, promoters or enhancers) across a genome in terms of a small set of co-occurring "basis" patterns. We will refer to these basis patterns as "codes". In contrast to previous approaches [16,17,25] we attempt to represent the unique "chromatin signature" at each locus as a weighted superposition of multiple basis patterns (*i.e.* each locus will be a linear combination of several codes with non-zero weights). We formulate the task of epigenetic code discovery in the framework of non-negative matrix factorization (NMF) [29,30]. This method transforms an input matrix *V* into two factor matrices *H* and *W*:

V≈WH

**Figure 1:**
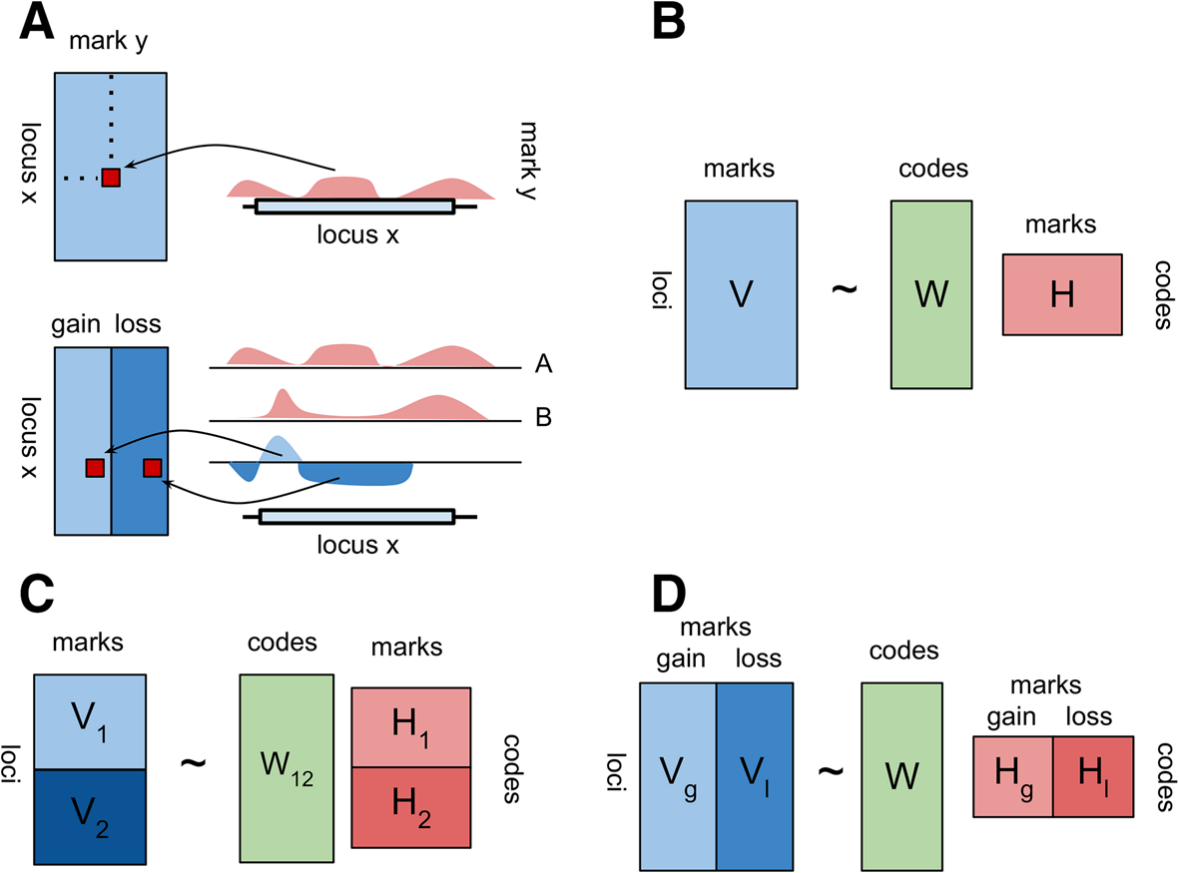
**NMF-based algorithms for epigenomic data.** **(A)** Schematic representation of the transformation of ChIP-seq data. (top) transformation of read counts into elements of the *V* matrix in the basic "absolute" mode. Each mark - locus combination is a single element in matrix *V*. Reads at each locus are summed. Columns of *V* are additionally scaled. (bottom) transformation of read counts from paired samples in "differential" mode. The differential signal is obtained by subtracting sample A coverage from sample B coverage after correction for sequencing-depth. Positive and negative area under curve is summed (integrated) into "gain" and "loss" scores. **(B)** NMF factorization in the "absolute" algorithm. *V* matrix is the same as the top of sub-panel **(A)**. **(C)** NMF factorization in the "discriminatory" algorithm. contains two classes of loci *V*_1_ and *V*_2_, which are used to derive two independent basis pattern matrices *H*_1_ and *H*_2_. All codes are concatenated and used to derive a single matrix *W*_12_. **(D)** NMF factorization in the "differential" algorithm. The *V* matrix corresponds to the bottom of sub-panel **(A)** and contains columns for the "gain" and "loss" of each mark.

**Figure 2:**
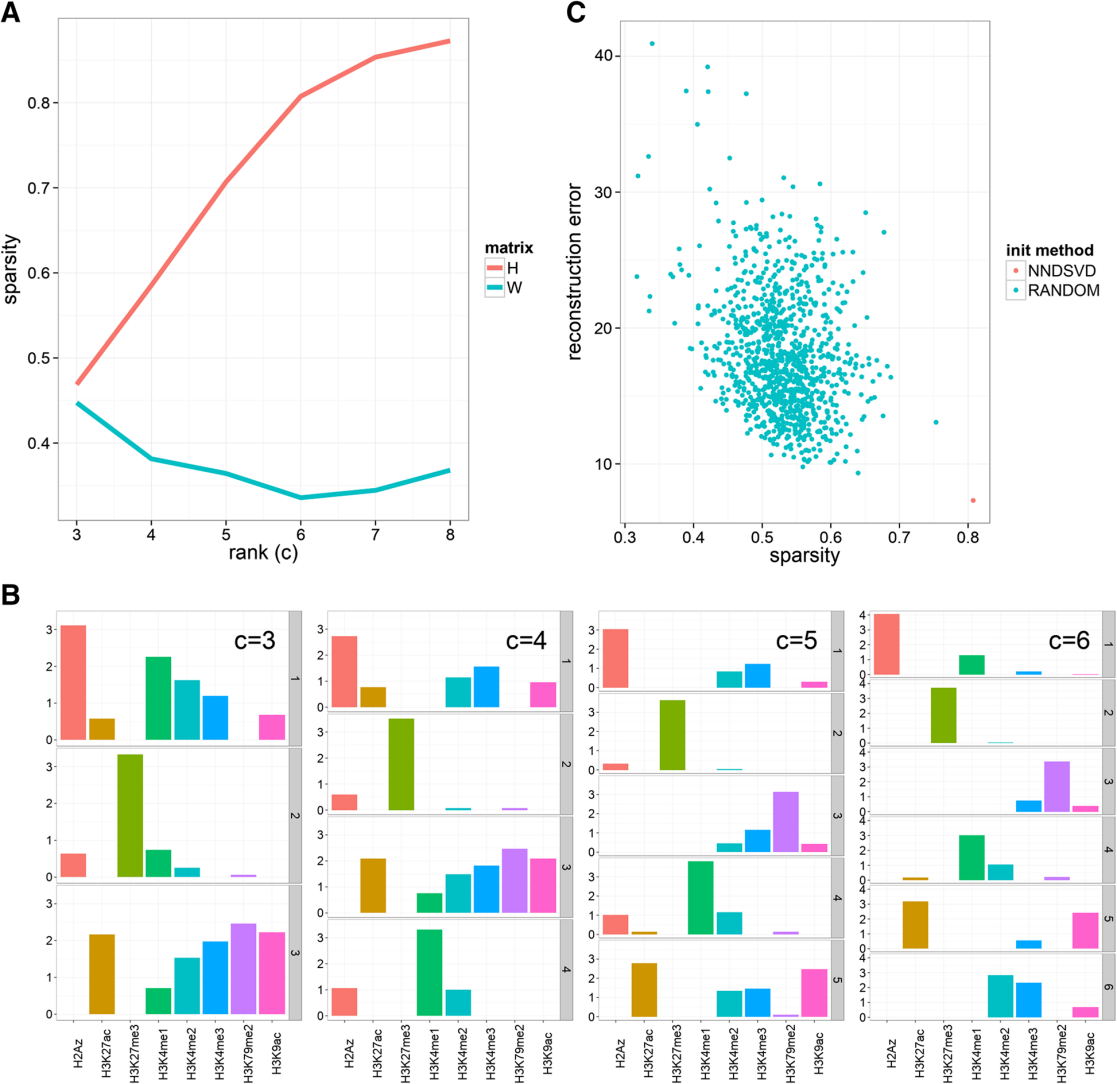
**Properties of NMF on epigenomic data.** **(A)** Average Sparsity of factor matrices *W* and *H* as a function of *c* calculated using Hoyer’s formula (see Results). **(B)** Approximate nesting of "basis patterns" for factorizations using different factorization ranks *c*. Nesting occurs if a lower rank basis vector is approximately derived by adding two higher rank basis vectors. **(C)** NMF reconstruction error and sparsity on 1000 random initializations of matrices *H* and *W*. NNDSVD – deterministic non-negative double singular value decomposition, random – uniformly (0 to 1 range) random non-negative matrices. The 1000 NNDSVDar randomizations find approximately the same factorization as NNDSVD and are not shown.

In the context of epigenomics *V* is a matrix of the observed "chromatin signatures". Each row of this matrix is an arbitrary user defined locus *e.g.* a region of 2 kbp flanking a transcription start site (TSS). Each column quantifies the level of a histone modification and is a function of the number of reads mapping to at least one base pair within this locus. *H* is a small matrix of sparse basis patterns, technically called basis vectors, which we refer to as codes, and *W* is a matrix of weights to reconstruct *V* using the codes in *H* (Figure 1B). Within a single basis pattern highly correlated input variables have positive values. We observed that for epigenetic marks the NMF algorithm yields a sparse matrix *H*. The resulting basis vectors in *H* are dissimilar and interpretable *e.g.* (Figure 2B). Unlike other matrix factorization methods, NMF is suitable for this particular task because it constrains both *H* and *W* to be non-negative. Given a factorization *V* ≈ *WH* we can assign code labels to genes by finding, for each gene, the code with the highest weight in *W*, which is analogous to "hard" cluster assignment in K-means [29,31].

The basic NMF procedure randomly initializes matrices *W* and *H* and minimizes the reconstruction error *V*-*WH* – difference between the actual and model output values of the epigenetic factor levels – by updating *W* and *H* using a projected gradient algorithm [32]. This algorithm finds only local optima that depend on the starting conditions, analogously to the common *K*-means algorithm. For the initialization of the NMF algorithm we propose to use the deterministic non-negative double singular value decomposition (NNDSVD) technique [33]. The NMF algorithm depends on a single parameter *c* - the rank of the factor matrices, which is the expected number of basis patterns. As with most unsupervised algorithms the choice of *c* is not straightforward. A large *c* results in sparse codes and few combinatorial interactions. Sparsity of the output is a prominent feature of NMF, but is further enhanced by additional constraints [34]. In our implementation the constraints are applied to the matrix *H* and thus favor combinatorial patterns of only few histone marks. To illustrate the sensitivity of NMF to initialization and the relative performance of NNDSVD for epigenomic data we compared the default factorization with a random initialization approach (Figure 2C). We found that this approach has the smallest reconstruction error (the objective function of NMF) and largest sparsity. Further, randomly initialized solutions tend to have a smaller reconstruction error if their *H* matrix is more similar to the NNDSVD solution (Additional file 2: Figure S1). Together, these results show that NMF output is sensitive to initialization. However, the NNDSVD approach yields a solution that outperforms even a large number of random runs.

We develop three complementary approaches which apply the NMF algorithm on epigenomic profile data for distinct tasks of prediction, classification and association: (1) absolute, (2) discriminatory and (3) differential (Figure 1). As shown in (Figure 1A-B), the absolute algorithm performs NMF on the quantified levels of epigenetic marks at one annotation class (*e.g.*, promoters). The discriminatory algorithm performs NMF on quantified levels of the same set of epigenetic marks at two classes of loci (*e.g.*, promoters and enhancers) (Figure 1C). As depicted in Figure 1D, the differential algorithm performs NMF on normalized differential epigenetic levels – gains and losses – between two cell lines or cell states (*e.g.* stem cells *vs* differentiated cells).

To construct *V* from genome-wide maps of multiple histone modifications, we individually quantify and scale "absolute" signals of epigenetic marks at each queried locus (Figure 1A top). Each row of the input matrix *V* represents scaled levels of epigenetic marks within a single locus. Some form of column normalization, or scaling, is usually necessary to account for the differences in magnitudes and dynamic ranges of histone modifications, and to reveal the patterns of interest [35]. By default we use a sigmoid function to normalize all signals to 0 to 1 range as this has been shown to accelerate and improve NMF [36,37].

Different classes of genomic regions, such as promoters and enhancers, show discriminatory epigenetic patterns [25]. Regulatory mechanism operating at distinct classes often have a unique epigenetic component, such as the activity of a specific chromatin remodeling complex (*e.g.*[38]). Thus, it is reasonable to assume that specific or enriched combinatorial patterns could discriminate between classes of sites. To identify such specific codes we propose the "discriminatory" algorithm (Figure 1C). In this mode we first apply the "absolute" algorithm at each set of *k* genomic regions separately and next reconstruct a single weight matrix. Specifically, we partition input matrix *V* into *k* sub-matrices *V*_*i*_, each of these matrices is independently factored *V*_*i*_ = *W*_*i*_*H*_*i*_, next we concatenate the *kH*_*i*_ matrices into a single matrix *H*. Finally the matrix *W* is obtained using non-negative least squares from matrices *V* and *H*. Intuitively, we first discover optimal codes for each class of genomic regions and next allow all codes to be used to describe the "chromatin signature" at each locus regardless of its class. If the epigenetic patterns at different classes of sites have the same latent structure, the discovered class-specific codes will be very similar or interchangeable. In both cases codes discovered for one class of sites will be useful to encode the epigenetic features of other classes of loci. On the other hand, if the latent epigenetic structure of the different region types is dissimilar, some of the discovered codes will be discriminatory and not useful to encode epigenetic features of other classes.

Histone modification levels are dynamic and are due to net changes in the activity of modifying enzymes called "writers" and "erasers" [39]. Relative to a second sample a locus might show "gain", "loss", or, if it is sufficiently large, both "gain" and "loss" of a histone mark. Although chromatin remodelling complexes often have multiple catalytic activities and substrate cross-reactivities [40], simultaneous changes to multiple marks at a subset of loci might suggest a shared regulatory mechanisms or function [41]. Therefore, we define basis patterns in the dynamic context as coordinated changes to histone modification levels. Analogously to the "absolute" and "discriminatory" cases, in "differential" mode (Figure 1D, Figure 1A bottom), mark levels are quantified within each query locus. However, because paired samples are typically sequenced to different depths, the mapped read counts are normalized using the DESeq algorithm [42]. Within each locus absolute signals are transformed into differential "gain-loss" scores (Figure 1A bottom). This approach results in twice the number of columns in *V* –two for each epigenetic mark. Histone modification levels are spatially auto-correlated. In "absolute" and "discriminatory" modes we rely on this property to calculate average enrichment levels within a possibly large locus. Much less is known about the auto-correlation of differential (subtracted) levels. Therefore we divide each locus into adjustable windows (default 100 bp). For each window paired ChIP signals are subtracted resulting in a net "gain" or "loss" of a histone modification. We obtain the final per-locus "gain" score by summing the windows with a net "gain", and the "loss" score by summing windows with a net "loss". If the differential signal is strongly auto-correlated most windows within a locus will show "gains" or "losses" and the whole locus will show only "gain" or "loss". A simple example shows that this is not always the case. If a peak is broadened it results in "losses" at the summit but "gains" at the slopes. Integrating over windows with sizes in the range of ChIP-seq resolution (hundereds of base pairs) allows us to differentiate these two cases. The per-locus columns are likewise scaled to the 0 – 1 range before entering the NMF method. The output is similar (Figure 1D): *H* contains basis "gain-loss" patterns *W* contains the weights associated with each pattern at each locus. The difference is that now rows in the matrix *H* correspond to patterns of correlated changes – not patterns of absolute levels.

### Algorithm properties

To illustrate important properties of NMF when applied to epigenomic data we ran the "absolute" algorithm on a relatively simple publicly available ChIP-seq data set. We analyzed 7 histone modifications and one histone variant (H2A.Z) mapped by the ENCODE project in the A549 adenocarcinomic alveolar basal epithelial cell line [43]. We focused on regions of TSS-proximal gene bodies since they contain epigenetic traces of transcription initiation and elongation, and prominently feature all probed marks.

To illustrate the dependence of *c* on the factorization we ran the "absolute" algorithm with all default parameters and scanned *c* values from 3 to 8. First, we quantified the average sparsity of matrices *H* and *W* using Hoyer’s formula [34] (Figure 2A). Hoyer’s sparsity takes on values between 0 (all vector elements equal) and 1 (single non-zero component). We observed that the sparsity of *H* increases linearly up to a knee-point at *c* = 6, whereas the sparsity of *W* is much lower and has a minimum at *c* = 6. This means that if *c* is (too) high the *H* matrix will contain many rows that have only a single mark with positive values. Matrix *W* contains weights that optimally use all codes to reconstruct the observed "chromatin signature" at each locus (rows of *V*). The relatively constant sparsity of *W* suggests that at most loci multiple basis patterns are used and superimposed (Figure 2A). An empirical property of NMF is that the higher-rank (large *c*) solutions are largely consistent with the lower-rank (small *c*) solutions. For example, in one study involving microarray clustering, higher resolution clusters are in general subsets of lower resolution clusters [30]. To illustrate this for basis patterns, we visualized matrices *H* for *c* = (3…6) (Figure 2B). This showed that codes obtained for higher *c* values are, in general terms, obtained by splitting one of the lower resolution codes into two. For example code 1 at *c* = 4 is split into code 1 and 5 at *c* = 5 while the latter is further split into code 5 and 6 at *c* = 6. This suggests that for NMF specifying a *c* which is (too) small yields a solution which is consistent with a higher (ostensibly correct) rank factorization. This type of stability is particularly useful when analyzing the hierarchical dependencies between histone modifications [44]. The lower-bound of *c* is determined by the diversity of histone modifications.

**Figure 3:**
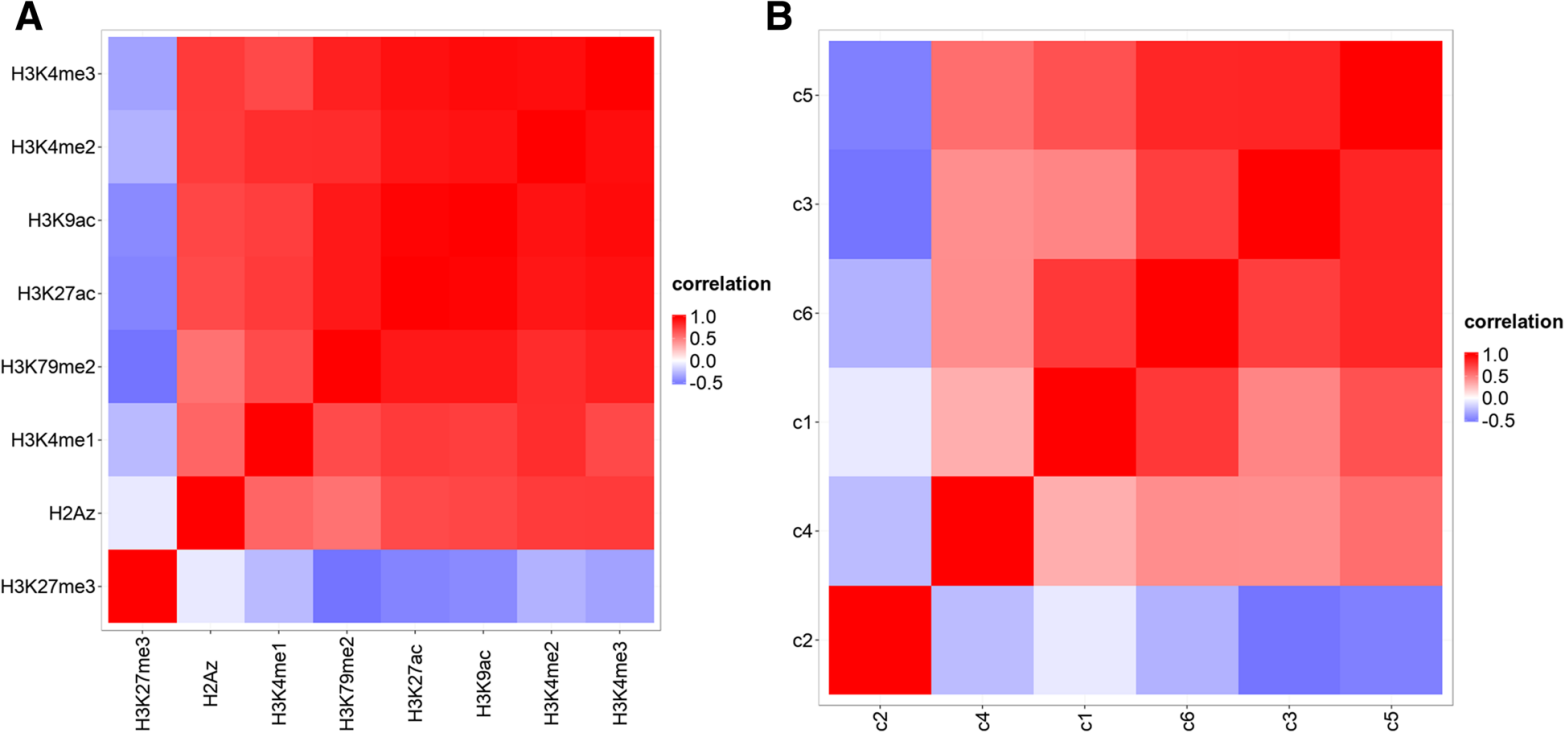
**Correlation of marks and codes within gene bodies.** Heat maps of Spearman’s rank correlation coefficients between epigenetic marks **(A)** and NMF basis patterns (codes) **(B)** in promoters of protein coding genes (marks were mapped in H1ESCs). The rows and columns of both heatmaps were sorted using Ward’s clustering [50] and the cosine metric [51].

The most important property we would like to highlight is that NMF basis patterns are less correlated than the input features. Correlation heatmaps are often used to reveal patterns of associations between histone modifications *e.g.*[45,46]. As exemplified in (Figure 3A) these heatmaps show typically little structure beyond the general split into marks associated with permissive or closed chromatin, which limits their interpretability. More importantly, the high correlation between histone modifications is problematic for regression and some classification methods [47]. High correlation among multiple variables in a regression model, referred to as multicollinearity, leads to poor interpretability of multiple regression slopes [48]. In particular, it is important to test for multicollinearity when attempting to use regression coefficients to assess the importance of variables (here levels of histone modifications and NMF basis patterns). We compared correlations of histone modifications to correlations between basis patterns (Figure 3B) and found that codes are remarkably less correlated. According to a rule of thumb variables that have correlation coefficients larger than 0.8 (Spearman’s rank correlation coefficient) should not be included together in a single model [49]. Only two pairs of NMF codes exceed this threshold and only a single code would need to be dropped. This is compared to 11 pairs of individual epigenetic marks that are exceedingly correlated. If all affected marks were dropped, the pruned model would contain only three independent variables. In Additional file 3: Figure S2 we show an analogous comparison with the difference that mark levels are calculated at promoters and in a different cell type (see Methods).

Another important feature of the NMF algorithm in absolute mode is the similarity of the *H* matrices across cell lines. In (Additional file 4: Figure S3) we show the *H* matrices from human embryonic stem (ES) cells (H1ESCs), myoblasts (HSMM blasts), and myotubes (HSMM tubes) derived from a set of 9 common epigenetic marks with *c* set to 6. In general, rows of each *H* matrix are in no particular order and equivalent codes obtained from two or more data sets have to be found using (for example) the Munkres assignment algorithm [52]. The NNDSVD approach initializes rows of matrix *H* using SVD eigenvectors and indirectly ranks basis patterns by their variance. This order is likely to be similar between different cell lines. We observe that the matrices are essentially the same for myotubes and myoblasts and only slightly different for H1ESCs. This suggest that the co-regulation of epigenetic marks is not drastically changing during differentiation. To get further insight on the complexity epigenetic patterns in terms of combinations of the *H* basis patterns we applied K-means clustering to the *W* matrix (Additional file 5: Figure S4). We clustered the *W* weight matrix corresponding to the *c* = 6 factorization from (Figure 2B). The majority of clusters (8) is dominated by at most 2 of the 6 codes, which means that for the majority of genes a simple weighted sum of two codes from (rows from *H*) is (globally) optimal to reconstruct relative levels of epigenetic marks. This should be contrasted with the hypothetical case, where most loci have highly variable and unique code weight patterns and the *W* matrix displays a second level of combinatorial complexity.

### Case studies

#### Regression Pol2 binding

In our formulation epigenetic patterns are quantitative *i.e.* each locus has a specific non-negative weight for each of the basis patterns. This enables us to quantitatively link the weights of codes to functional or biochemical properties of the underlying loci. To illustrate this we tried to predict levels of Pol2 binding at promoters of protein-coding genes in human embryonic stem cells (H1ESC). We compared ridge regression models, which either included basis patterns (code-based) or individual histone marks (mark-based) as independent variables. Levels of histone modifications and Pol2 were calculated within 5k kbp window centered at the TSS.

**Figure 4:**
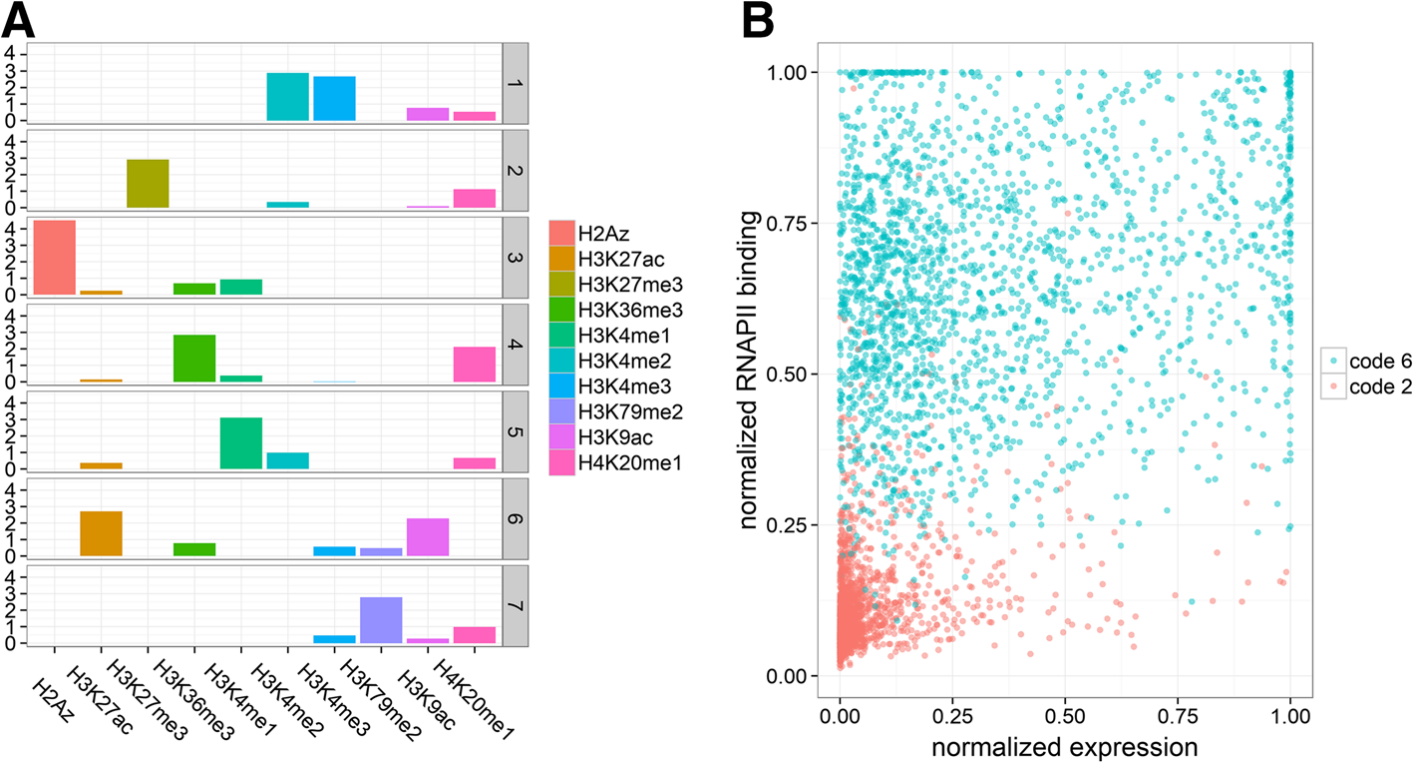
**Basis patterns at promoters and their role in Pol2 recruitment.** **(A)** Basis patterns associated with promoter-regions in ES cells. Graphical representation of the *H* factor matrix of the "absolute" algorithm. Rows correspond to basis patterns (codes), columns to epigenetic marks. The height of each bar is equal to the loading of a mark on a code. The codes are sparse *i.e.* most values are very close to zero. Most marks have significant loading (by visual inspection) in one or two codes with the exception of H4K20me1. **(B)** Codes in the Pol2-expression plane. Each dot represents a promoter of a protein-coding gene. The Y-axis represents normalized levels of Pol2 binding; the X-axis is the normalized expression level the associated gene. Each genes was assigned a label of the code with the highest weight. Genes assigned to codes 2 and 6 are shown.

To obtain the code (*H*) and weights (*W*) we applied the algorithm in "absolute" mode on 10 histone modifications with default parameters and *c* = 7. The discovered codes are shown in (Figure 4A). As expected (see Properties), we found that codes are not significantly correlated and that all of them should be included in the multiple regression (Additional file 3: Figure S2B). On the other hand, six pairs of individual epigenetic marks are exceedingly correlated (Additional file 3: Figure S2A). The primary reason why highly correlated variables should not be included in a multivariate model is that their beta regression coefficients become unreliable both in magnitude and sign, and thus their biological or physical role is difficult to interpret. This suggests that at least three marks out of (H3K4me2, H3K4me3, H3K9ac, H3K27ac, H3K79me2) should be dropped. Unfortunately, it is not *a priori* known which ones (an alternative method to establish variable importance and mitigate effects of multicollinearity is to inspect the output of penalized regression). The mark-model’s performance dropped significantly from *r*^2^ = 0.85 to *r*^2^ = 0.70 when we kept two marks that are known to be associated with both recruited (H3K4me3), and actively transcribing (H3K79me2) Pol2 [20,53]. Although the code-based regression includes fewer independent variables it has almost the same performance (both *r*^2^ = 0.85) as the mark-based model (Table 1). An inspection of the regression slopes (Table 1) and code values (Figure 4A) reveals that high weights of code 6 (H3K9ac/H3K27ac) and code 1 (H3K4me2/H3K4me3) are most positively associated with Pol2 levels, which fully confirms a recent study [20]. Due to multicollinearity, coefficients of the mark-based regression are not reliable to rank variable importance. For example, the negative beta for H3K4me3 is inconsistent with numerous reports that link H3K4me3 to Pol2 binding and transcription [54], which idictates overfitting although a penalized regression approach was employed.

**Table 1:** Parameters and performance of mark-based and code-based ridge regression models

**Code-based**		**Mark-based**	
code	beta	mark	beta
code 1	13.48	H2AZ	-1.33
code 2	1.64	H3K27ac	1.28
code 3	-24.98	H3K27me3	-0.33
code 4	12.63	H3K36me3	0.73
code 5	6.55	H3K4me1	-0.17
code 6	32.24	H3K4me2	1.17
code 7	-2.28	H3K4me3	-0.80
		H3K79me2	-0.63
		H3K9ac	3.41
		H4K20me1	0.73
R2	0.85	0.85	
MSE	0.01		0.01

Ridge regression of Pol2 levels in promoters of protein coding genes. beta – regression coefficients; MSE – mean squared error.

To differentiate active transcription from promoter-proximal Pol2 pausing we assigned each gene to the basis pattern with the highest weight (see Formulation) and plotted genes from select codes in the gene expression – Pol2 level plane. This projection revealed that genes from code 6, featuring most prominently high levels of H3K9ac and H3K27ac, have all moderate to high levels of Pol2. In contrast, genes associated with code 2, which is dominated by H3K27me3, have uniformly low levels of Pol2. Remarkably, high levels of these activating acetylations are not significantly correlated with gene expression, while H3K27 tri-methylated genes tend to be expressed at a low level. This suggests that high levels of H3K27me3 are incompatible with Pol2 binding, and that high levels of Pol2 are associated with K3K9ac and H3K27ac at gene promoters but not necessarily high gene expression.

In this example we have shown that quantitative weights of the "absolute" basis patterns can be used instead of individual histone modifications levels as independent variables in the prediction of Pol2 binding. The code-based model had equal performance to the mark-based regression, but included a smaller number of independent variables and alleviated problems of multicollinearity. Hard assignment of genes to codes allowed visualization of the regulatory differences in Pol2-recruitment and active transcription.

#### Classification Pol2-bound enhancers *vs.* promoters

Polymerase II (Pol2) is known to localize both at promoters and within intragenic regions. In H1ESC preferential association of Pol2 was observed for promoter-distal sites enriched for p300, H3K4me1, and H3K27ac [55]. Genes in the vicinity of these regulatory regions showed increased expression levels, while genes that were activated during differentiation gained Pol2 at close enhancers [56]. In differentiated cells Pol2 levels at enhancers have been shown to change in response to stimuli and to be associated with H3K4me3 and bidirectional transcription [57]. These findings established that enhancers actively engaged in transcription are occupied by the polymerase. The chromatin patterns of this class of enhancers show relatively high levels of H3K4me3 and are more similar to patterns at promoters of protein coding genes. We decided to test whether Pol2-bound enhancers and Pol2-bound promoters can be distinguished based on levels and multivariate patterns of epigenetic modifications.

In the same line of human embryonic cells we divided Pol2-enriched regions into two classes. The promoter-proximal class was defined as 2 kbp regions centered on an Pol2 peak, that overlapped any GENCODE annotated TSS site. All remaining 2 kbp sites centered on an Pol2 peak were classified as promoter-distal. We performed the analysis using all promoter and enhancer regions, but found the classification was relatively trivial because the largest Pol2 peaks are preferentially associated with promoter regions [55]. Thus, we challenged the classification algorithm by rerunning the analysis excluding the top 20 percent of peaks *i.e.* those with a very high p-value of 1e-25. First, we compared the overall distribution of histone modifications at the promoters and putative enhancers.

We found that some marks showed relatively similar levels (Additional file 6: Figure S5). As expected we found substantial H3K4me3 levels at Pol2-bound putative enhancers. Strikingly, levels of H3K4me1 and H3K4me2, which are often associated with poised or active enhancers, were markedly higher in TSS-proximal sites. On the other hand, H3K27ac, which is associated with permissive chromatin, and H4K20me1/H3K79me2, which are associated with transcriptional elongation, had similar levels at both classes of sites. In agreement with recent discoveries, we found that a significant portion of intragenic Pol2 sites occurred within "poised" enhancers that were enriched for H3K27me3 (Additional file 6: Figure S5). Notably, while there were some informative level differences, the distributions significantly overlapped for the majority of marks.

To discriminate enhancer from promoter regions we first built a series of logistic regression models. The simplest models ("zero-order" models [23]) included only a single independent variable (*i.e.* the normalized level of a single histone modification). These zero-order correlations directly measure the shared variance between two variables, since they reflect the amount of variance in the binary outcome variable that is explained by a single continuous predictor. In addition a "multivariate" model was built that included levels of all marks as predictors. An analogous set of zero-order and multivariate models was built using NMF codes. This new set of models differed from the previous in that they used weights from the *W* matrix rather than levels of individual marks to perform the classification. We applied the "discriminatory" algorithm and discovered optimal codes for enhancers and promoters independently (Figure 1C, Formulation). Intuitively, we attempt to identify codes that are useful to encode histone modification levels at enhancers, but not promoters (or *vice versa*). We combined all codes into matrix *H* and re-derived the weight matrix *W*. Therefore, weights for certain codes should discriminate between promoters and enhancers.

**Figure 5:**
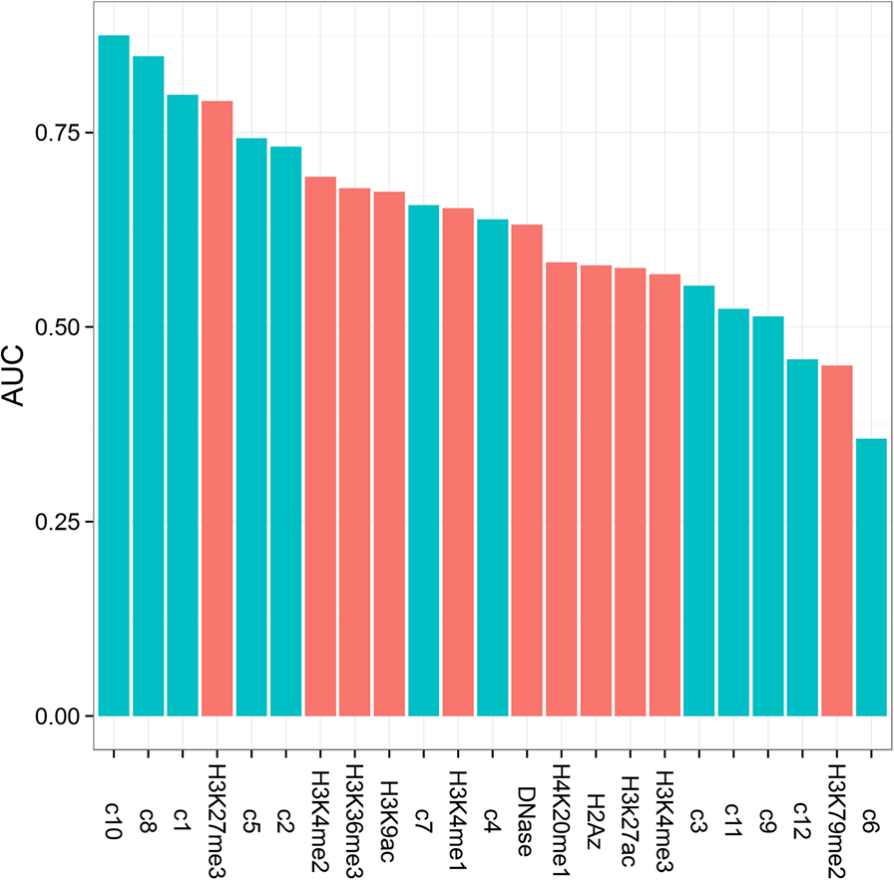
**Classification performance of individual marks and codes.** Classification performance between TSS-proximal and TSS-distal Pol2-bound sites estimated as the area under the ROC. Classification on individual marks or codes was done using *L*_2_ penalized logistic regression and all models were trained using 10-fold cross validation and the model with the highest performance was evaluated on a holdout set of 20 percent observations. Codes and their weights were obtained by the "discriminatory" algorithm.

We trained all models using 10-fold cross-validation and evaluated model performance on 20 percent of the observations never used for training (Methods). We found that both multivariate models had very good performance (Additional file 7: Table S1) as judged by the Matthews correlation coefficient (MCC), whereas among zero-order models only some code-based regressions showed good performance (Area Under the receiver operating characteristic (ROC) Curve (AUC) in Figure 5, MCC in Additional file 8: Table S2). The multivariate code-based model outperformed the mark-based model in both performance measures and achieved an almost perfect score AUC 0.97 (Additional file 7: Table S1). The majority of the mark-based zero-order models had similar and average performance, whereas the AUC scores of zero-order code-based models were highly variable (Figure 5). Single code models either outperformed all histone modifications (with the exception of H3K27me3) or were close to the performance of random assignment (Figure 5). Importantly, the two best single-code regressions (c10 and c8) had significantly better performance than all individual histone modifications including H3K27me3. While code 10 contained H3K27me3 together with H2A.Z, code 8 was dominated by marks associated with elongation including H3K36me3 and H4K20me1. This shows that the codes had discriminating power beyond that of the best mark (H3K27me3 in this case).

To assess the relative importance of independent variables in multivariate regression it is important not to rely only on regression coefficients [23]. One approach is to compare the ranking and signs of variables from zero-order and multivariate models. We found that mark-based logistic regressions have incongruent slope estimates. For example beta coefficients of three marks change signs between the two models. Also the ranking of the beta coefficients are not even approximately maintained and do not track model AUCs (data not shown). In the mark-based case it was difficult to ascertain which histone modifications discriminate enhancers from promoters. In contrast regression on codes yields models that are easier to interpret. Specifically, codes with large zero-order coefficients were also relatively important in the multivariate model, which largely maintained the rank-order of variables (Additional file 9: Figure S6). Also, codes with the largest multivariate coefficients consistently showed the best zero-order predictive performance (Figure 5B). Several codes have very small zero-order coefficients and AUCs, but relatively large multivariate slopes. Likely, these codes are not important and could be dropped from the multivariate model.

**Figure 6:**
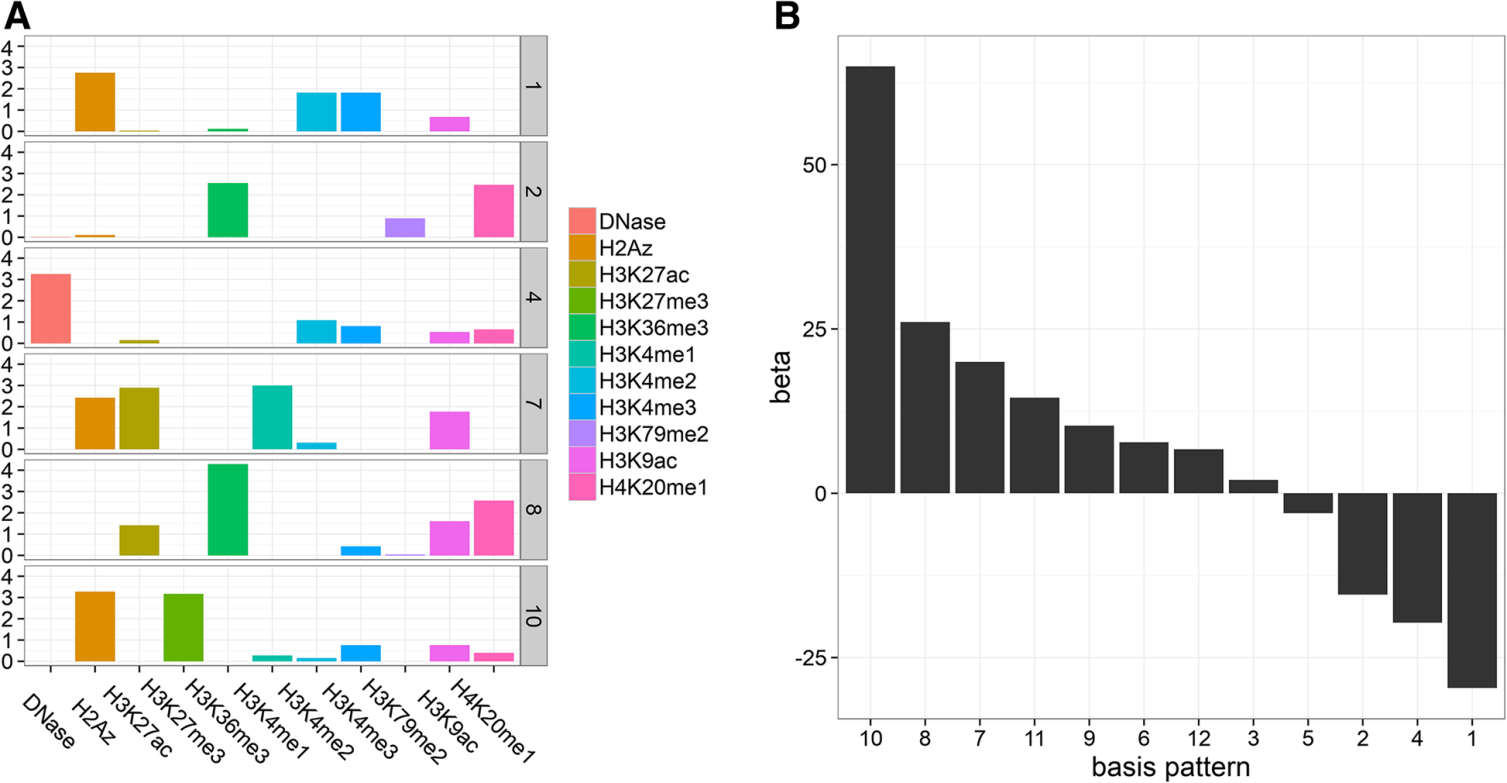
**Code-based classification of Pol2-bound promoters and enhancers in ES cells.** Parameters of a multivariate penalized logistic regression model using code variables. Codes and their weights were obtained by the "discriminatory" algorithm. **(A)** Basis patterns associated with the six coefficients with the largest magnitude **(B)** Coefficients of all basis patterns, where positive and negative values indicate propensity towards TSS-proximal and TSS-distal regions, respectively. In total 12 codes were trained: 6 on TSS-proximal and 6 on TSS-distal regions. The weights in *W* were reconstructed using all 12 basis patterns (Figure 1C).

The code-based approach allowed us to identify which patterns of histone modifications discriminate between Pol2-bound promoters and enhancers (Figure 6 and Additional file 10: Figure S7). Most strikingly, we found that promoters and enhancers were separated by codes with high levels of H2A.Z. In the context of promoters H2A.Z is linked to H3K4me2 and H3K4me3. At enhancers H2A.Z frequently co-occurs with H3K27me3 or in a complex pattern with H3K4me1, H3K9ac, and H3K27ac. This explains why the variant on its own is unable to differentiate sites (Figure 5, Additional file 9: Figure S6A). Recent findings on the functional and mechanistic roles of H2A.Z allow us to give plausible interpretations of the codes: At the TSS H2A.Z levels and close positioning have been shown to positively correlate with gene expression and Pol2 occupancy [58], also high levels of H3K4me2, and in particular H3K4me3, are generally associated with active promoters. Hence, code 1 is likely associated with genes that are transcriptionally active in the ESC state. H2A.Z has been shown to be associated both with poised and active enhancers in ESC [59]. It has been proposed to act as a general facilitator of genome accessibility due to its role in the maintenance of pluripotency and differentiation [39]. The two H2A.Z-loaded enhancer codes (10 and 7) seem to reveal this context dependent role of H2A.Z. In code 10, H2A.Z is in a repressive context with H3K27me3 and presumably identifies enhancers poised for expression during differentiation while in code 7 H2A.Z co-occurs with permissive acetylations and the base-line enhancer mark H3K4me1 [60,61]. Code 7 highlights features of enhancers active in the pluripotent state.

Surprisingly, both enhancers and promoters, are associated with codes dominated by histone modifications associated with transcription elongation. At promoters this code is very sparse and contains non-zero values for H3K36me3, H4K20me1 (both high), and H3K79me2 (low). At putative enhancers the code is slightly different as it does not contain H3K79me2, but includes H3K9ac and H3K27ac at low levels. Recently it was shown that H3K79me2 is most enriched at 5’ ends of genes, slightly downstream of H3K4me3, but before the classic elongation associated mark H3K36me3 [62]. Thus, it is expected to occur at promoter proximal Pol2-bound regions. To the contrary, active enhancers are sometimes found in introns of transcribed genes. This dependency between active transcription and activation of enhancers in the gene body appears to be captured via code 7.

In total, these results suggest that the discovered basis patterns capture dependencies between marks that discriminate Pol2-bound enhancers or promoters. The factorization approach successfully de-correlated epigenetic marks, which resulted in an interpretable multivariate classification model. Further, the discovered codes are consistent with known epigenetic mechanisms and features that regulate Pol2-dependent transcription in pluripotent cells.

#### Gene set enrichment analysis

In the previous analysis we have compared "absolute" levels of histone modifications at multiple classes of loci to discover patterns of co-occurring marks that discriminate among them. Somewhat analogously, histone modification levels can be compared between two experimental conditions. Intuitively, the idea is that patterns of co-occurring changes to mark levels could be used to identify loci that are subjected to coordinated epigenetic regulation. Differentiation is a highly regulated process and specific reprogramming mechanisms could result in similar epigenetic changes at functionally related genes. In other words, genes that share combinatorial patterns of changes could have some common molecular functions or participate in related pathways.

**Figure 7:**
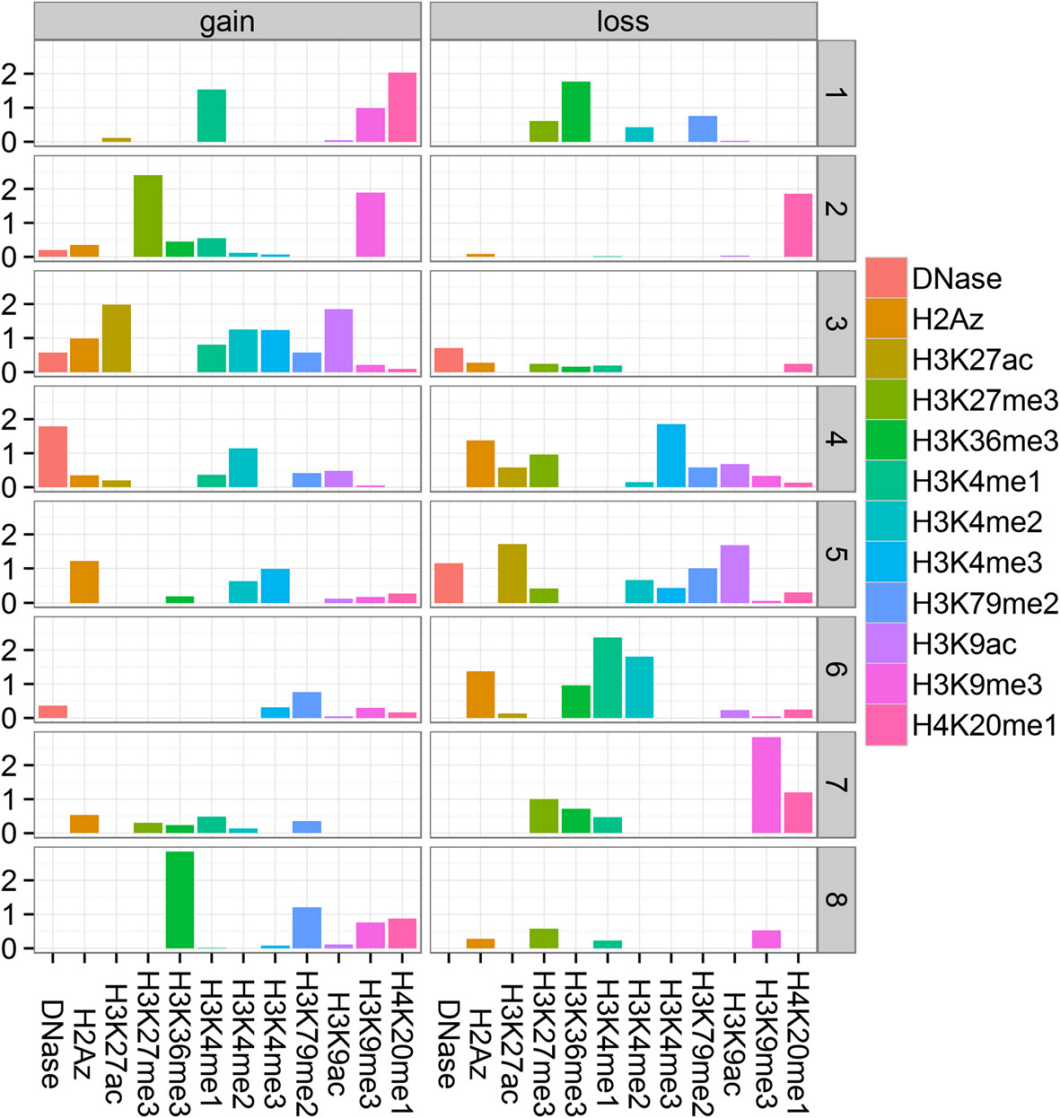
**"gain-loss" codes of epigenetic reprogramming during differentiation.** Graphical representation of the *H* factor matrix of the "differential" algorithm. Rows correspond to "gain-loss" basis patterns, columns to both gains and losses of epigenetic marks. The height of each bar is equal to the loading of a mark gain or loss on a code. Columns are grouped based on the direction of epigenetic change ("gain" or "loss"). Within a single code it is rare to observe both a significant "gain" and "loss" of the same mark. Most mark changes have significant loading in two codes with the exception of H3K9me3 gain and H3K27me3 loss. The majority of patterns combines "gains" and "losses"of multiple marks.

To test this hypothesis we applied the "differential" algorithm (see Formulation) to histone modification data in myoblasts and myotubes. The alignment of myoblasts into myotubes represents an important step in myogenesis and is an example of differentiation. Thus, by comparing the epigenetic state of myotubes to myoblasts we hope to discover patterns of epigenetic changes that are associated with genes that undergo coordinated epigenetic reprogramming during myogenesis [63]. We obtain basis "gain-loss" patterns from the observed epigenetic changes at promoters of protein coding genes (see Formulation and Methods). The number of codes represents a significant reduction from the 24 input variables – the 12 gains and 12 losses of epigenetic marks (Figure 7). Still, the codes are relatively sparse and most variables take significant values in only a small number of codes. It should be noted that the codes are not gain- or loss-specific and gains of certain marks are linked to losses of other marks. For example, one pattern highlights H3K4me3 and H2A.Z loss linked to an increase in DNase I accessibility and gain of H3K4me2 (code 4, Figure 7).

We tested whether any of the basis patterns are enriched for Gene Ontology (GO) terms, biochemical pathways, or experimental molecular signatures [64]. Specifically, we evaluated the strength of positive association between the weights of each code at each locus with functional annotations of the underlying loci (see Methods). We have chosen the random-set method [65] to quantify the enrichment, but other methods including the Gene Set Enrichment Analysis (GSEA) [66] and the Fisher’s exact test are equally applicable. Surprisingly, we found only one statistically significant (after False Discovery Rate (FDR) correction) GO term: code 5 was found to be associated with genes involved in the cell cycle (p-value: 6.20e-17). Several codes are significantly associated with specific pathways (Table 2). Broadly, membrane proteins and specifically G protein-coupled receptors (GPCR) have increased weights of the repressive code 2; code 3 is linked to genes involved in transcription; splicing and translation; finally, genes involved in the cell cycle are, again, associated with higher levels of code 5. These pathways are critical to myogenesis. During differentiation myoblasts exit the cell cycle and increase protein synthesis to expand the myofibrillar muscle cell compartment [67]. Fully mature muscle cells express tens of different GPCR receptors [68]. Many of which have been shown to enable muscle function by regulating growth, contractility, and glucose uptake. However, the genes associated with code 2 (neuroactive (p: 1.57e-43) and olfactory (p: 2.09e-17) GPCRs) are transcriptionally silenced as cells transition from myoblasts to myotubes. Among molecular signatures we found over 350 highly enriched terms (p: 1e-10) (Additional file 11). The most significant association was between targets of E2F4 and the "gain-loss" code 5 (p: 8.54e-156). E2F4 is a transcriptional regulator with a specific role in the repression of cell-cycle genes and ability to recruit HDAC1-containing co-repressor complexes [69]. It is notable that the most prominent feature of code 5 is the loss of H3K9ac and H3K27ac. Although HDACs have relatively low substrate specificity, which is dependent on their co-factors, HDAC1 has been recently implicated in the specific deacetylation of H3K9 [70]. A prediction based on this analysis is that H2A.Z levels increase at cell cycle genes repressed during myoblast differentiation. Taken together these results suggest that during myogenesis distinct patterns of net gains or losses of epigenetic marks are associated with functional classes of genes.

**Table 2:** Statistical association of epigenetic remodelling patterns and molecular pathways during myoblast differentiation

**Code**	**Pathway identifier in MSigDB**	**p-value (FDR)**
code 2	REACTOME_GPCR_LIGAND_BINDING	1.25E-48
code 2	REACTOME_CLASS_A1_RHODOPSIN_LIKE_RECEPTORS	5.80E-39
code 2	KEGG_NEUROACTIVE_LIGAND_RECEPTOR_INTERACTION	1.57E-34
code 2	REACTOME_PEPTIDE_LIGAND_BINDING_RECEPTORS	2.00E-32
code 2	KEGG_OLFACTORY_TRANSDUCTION	2.09E-17
code 2	KEGG_CYTOKINE_CYTOKINE_RECEPTOR_INTERACTION	4.39E-12
code 3	REACTOME_GENERIC_TRANSCRIPTION_PATHWAY	1.18E-11
code 3	REACTOME_TRANSCRIPTION	3.38E-08
code 3	REACTOME_NONSENSE_MEDIATED_DECAY_ENHANCED...	1.41E-06
code 3	REACTOME_METABOLISM_OF_PROTEINS	3.68E-06
code 3	KEGG_SPLICEOSOME	3.68E-06
code 3	REACTOME_MUSCLE_CONTRACTION	6.62E-06
code 4	REACTOME_CELL_CYCLE_MITOTIC	1.88E-07
code 5	REACTOME_CELL_CYCLE	2.28E-68
code 5	REACTOME_CELL_CYCLE_MITOTIC	4.19E-48
code 5	REACTOME_TRANSCRIPTION	7.90E-35
code 5	REACTOME_MITOTIC_M_M_G1_PHASES	1.10E-31
code 5	REACTOME_DEPOSITION_OF_NEW_CENPA_...	1.69E-28
code 5	KEGG_SPLICEOSOME	2.32E-27
code 6	REACTOME_CELL_CYCLE	2.15E-06
code 8	REACTOME_GENERIC_TRANSCRIPTION_PATHWAY	1.64E-09

For each of the "gain-loss" basis patterns only the most significant (FDR-corrected) terms are shown for each code. Pathway gene annotations and identifiers are from MSigDB, but are originally sourced from KEGG and Reactome. Codes that did not have any significant terms at the 1E-6 level were omitted.

**Figure 8:**
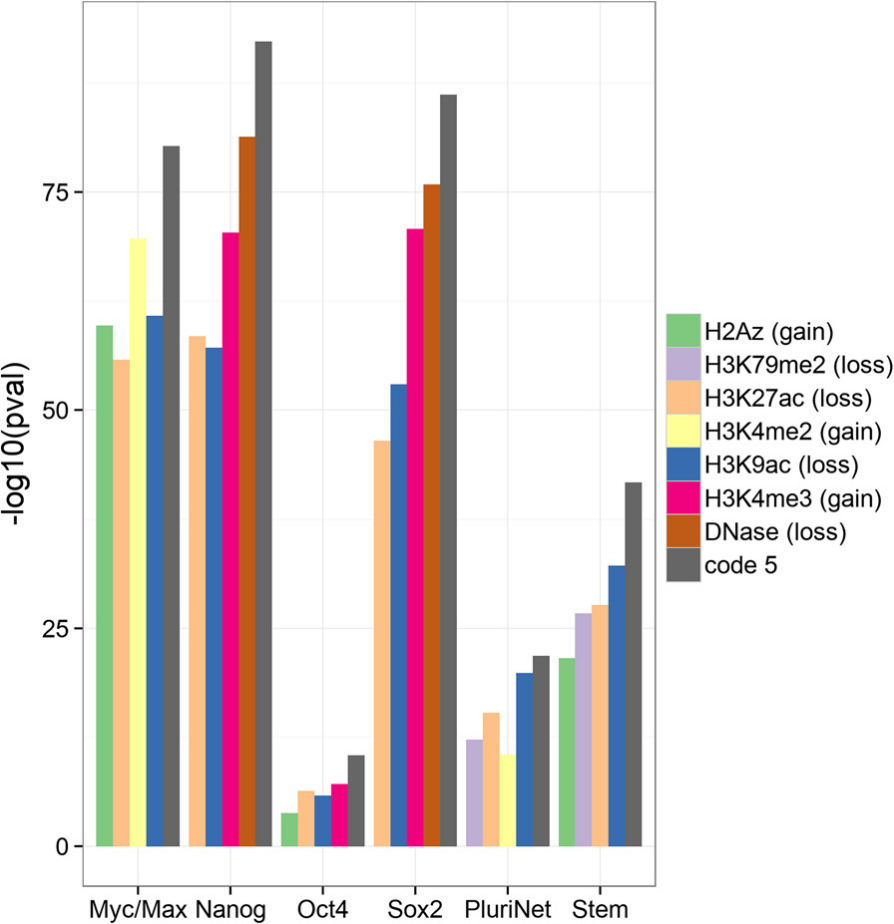
**Epigenetic reprogramming of the pluripotency network.** Association of histone modification changes and NMF codes with genes involved in pluripotency. Myc/Max, Nanog, Oct4, Sox2 are shorthand for experimental targets of these TFs in human embryonic stem cells. PluriNet are genes involved in the protein-protein interactions networks discovered in pluripotent cells. Stem are genes up-regulated in embryonic stem cells shared between mouse and human. The five marks or codes most significantly (p-value) associated with each group of genes are shown.

Myoblasts are embryonic progenitor cells with myogenic potential. They are more differentiated than ES cells, but markedly less than myotubes. Expression of pluripotency factors can either abrogate differentiation into myotubes or elicit reprogramming of myoblasts into induced pluripotent stem cells (iPSC) [71,72]. We hypothesized that targets of pluripotency factors which are silenced during differentiation will share an epigenetic signature of their repression. We found that experimental targets of several pluripotency factors, including MYC (c-Myc), NANOG, POU5F1 (Oct4), and SOX2, are all strongly associated with the repressive "gain-loss" code 5 (Additional file 11). Further significant enrichments were observed for other "pluripotent" gene categories including the protein-protein interaction network shared among pluripotent cells (PluriNet) [73], and the core ESC-like module [74], which includes genes coordinately up-regulated in a compendium of ESCs. We decided to test whether these enrichments are due to the specific combinatorial pattern of gains and losses captured by code 5, or alternatively could be explained by any of the individual marks (Figure 8). We found that each category of pluripotent genes is more strongly associated with code 5 than with any of the single epigenetic marks. Further, the association of other epigenetic marks within the six categories was inconsistent and no single mark could be chosen as a strong proxy for code 5. This suggests that promoters of genes that maintain the pluripotent state are epigenetically silenced in a coordinated way and that this is captured by one of the "gain-loss" epigenetic "codes". Other patterns of silencing *i.e.* codes 4 and 6 which display loss of H3K4me3/H2A.Z and H3K4me2/H3K4me1/H2A.Z, respectively are not consistently associated with the pluripotency categories.

## Discussion

We have introduced a new computational technique to discover combinatorial patterns of histone modifications. At its core this method relies on non-negative matrix factorization (NMF) to separate the complex "chromatin signatures" at genomic loci into small basis patterns we refer to as codes. These simple parametrizations of the data reveal frequently co-occurring marks, which could potentially be read by multivalent chromatin complexes, or represent differential signatures of coordinated epigenetic reprogramming. Most importantly, the application of NMF results in dimensionality reduction and de-correlation, but maintains the quantitative aspect of epigenetic mark levels.

NMF is one among many matrix factorization algorithms. Results from alternative methods are different due to the difference in the imposed factorization constraints and objectives. Principal component analysis (PCA) constrains *H* to be a set of orthonormal vectors; vector quantization (VQ), which is equivalent to K-means, constrains *W* to contain vectors with one non-zero value; while NMF imposes that *W* and *H* are non-negative. These constraints result in fundamentally different outputs. PCA favors global reconstruction, which means that every element in *V* is reconstructed through complex cancellations of positive and negative values in *W* and *H*. PCA allows basis vectors (principal component, PC) in *H* to be ranked by importance. The reconstruction error increases when the least important PC is omitted, but the "coarse" global features of input data are preserved. On the other hand NMF basis vectors cannot be dropped, since it would result in the loss of important parts of (a subset of) the reconstructed vectors.

While PCA dimensions do not resemble any particular data point or combination of data points, NMF basis vectors can be readily interpreted as patterns of frequently co-occurring histone modifications. Only a small number of these codes is sufficient to reliably reconstruct the observed "chromatin signatures" at thousands of loci and, as shown by our analyses, to preserve, or even boost, biological information. Although with respect to the mean squared error (MSE) PCA is theoretically optimal for reconstruction, NMF can perform better for classification [75] or recognition [76]. In some sense NMF returns results that are in between PCA and VQ. In VQ each data point is locally approximated by a single cluster centroid, PCA uses all available components, while in NMF typically few, but not all, basis vectors are required to represent a single data point. If the goal is to assign loci to epigenomic states a form of clustering is preferred as cluster centroids are often intuitively understood. PCA will perform best if the number of histone modifications is large but one desires only few basis vectors (principal components). As illustrated in this paper NMF basis vectors perform well in supervised machine learning. An alternative and analogous approach, known as principal components regression, is to use weights of principal components instead of weights of NMF basis vectors. An advantage of NMF is the physical interpretability of the its basis vectors. Conversely, the optimal reconstruction error of PCA might be important for very simple models.

## Conclusions

We have shown that NMF applied to epigenetic marks yield sparse codes with an important nesting property. Further we have demonstrated the benefits of using codes over individual marks in predictive modeling of Pol2-binding. In particular, dimensions obtained from NMF are less correlated than the individual marks, and problems resulting from multicollinearity are alleviated. In addition we developed two variants of the basic algorithm which extended its applicability to multiple classes of genomic regions and paired experimental samples. We have shown the excellent performance of codes for the classification of Pol2-bound enhancers and promoters. The most discriminatory codes highlighted the context-dependence of H2A.Z, which is consistent with current knowledge on the role of this histone variant in the regulation of transcription in ES cells [59]. To showcase the algorithm for paired experimental samples, we analyzed chromatin remodeling during myogenesis. We established that genes from pathways involved in protein synthesis (anabolism), the cell cycle, and signaling from G protein-coupled receptors show unique patterns of chromatin activation or silencing. Finally, we were able to show that target genes of pluripotency factors are also associated with the same chromatin remodeling pattern [77].

In summary, we have introduced a general NMF-based approach to represent combinatorial patterns of epigenetic marks as quantitative variables. We have shown the utility of this representation for predictive modeling, supervised machine learning and gene set analysis. Hence, this technique is complementary to more descriptive methods aimed at "chromatin pattern" discovery such as genome-wide segmentation and clustering.

## Methods

### Implementation

All three variants of the presented NMF-based algorithm are provided as the epicode open-source software package. The software provides all that is required to discover basis patterns from aligned sequencing data and sets of user-provided reference regions. Epicode provides three modes of operation: "absolute" and "discriminatory" and "differential". In the "absolute" mode the user is expected to provide a set of genomic loci (in a UCSC Browser Extensible Data (BED) file) of interest and aligned sequencing data for a single experimental condition (in Binary sequence Alignment/Map (BAM) files). The regions can be global such as promoters of protein coding genes or specific subsets *e.g.* "putative enhancers of expressed miRNAs". The input sequencing data are typically histone modifications mapped in a single cell line and experimental condition. In the "discriminatory" mode the user provides two sets of loci *e.g.* enhancers and promoters. The "differential" mode requires a single set of genomic regions, but two sets of sequencing data, which correspond to the same marks mapped in two conditions or cell lines.

We have implemented epicode as a Python 2.7 software package, and also provide a command-line executable. The code should run on UNIX-like operating systems and has been tested on Linux (Arch, RHEL 6). Dependencies: several Python packages are required by epicode, including NumPy [78], SciPy [79], scikit-learn [80], and pysam. Input formats: the tool is designed to work with standard file formats. Reference genomic sites are expected in the BED6+ file format [81]. Sequencing data is read from coordinate sorted BAM files [82]. Output: Results are reported in a machine-readable tab-delimited file format. Scripts in the R language are provided to generate publication quality figures from one of the output files. The current implementation of Epicode is IO-bound meaning that the majority of time is spent in reading the BAM files. The factorization takes typically less than 5 minutes on a single Intel(R) Xeon(R) CPU E5-1620 0 3.60 GHz core. Reading the BAM files takes up-to 30 minutes using four cores and strongly depends on the hard-drive speed. 6. URL and license for software should be mentioned in manuscript. The software is freely available (MIT license) at https://github.com/mcieslik-mctp/epicode.

Throughout the manuscript epicode has been used with all default parameters (as of version 1.0), with the exception of the "differential" algorithm for which a step of 50 was chosen.

### Data normalization

To construct matrix *V* from sorted BAM files, individual reads are counted within regions (lines) of the user provided BED files. Each read that overlaps the target region is counted towards that region. All columns (marks) of *V* are scaled from 0 to 1 using a sigmoid function such that the mapping is approximately linear up to the 95th percentile [36].

$$\hat{x}=2/(1+e^{\left(-2x/u\right)})-1$$

Here, *u* is the 95th percentile of the values in vector *x* and $\hat{x}$ is the scaled vector. The scaling is done before factorization. In "differential" mode enrichment signals (Figure 1D) are windowed and corrected for sequencing-depth (*i.e.*, normalized) using the provided Python implementation of the DESeq algorithm [42]. After subtraction the window scores are summed to overall "gain" (positive integral) and "loss" scores (negative integral) for each locus. "Gain-loss" scores are likewise sigmoid scaled.

### Enrichment analysis

Associations of functional gene sets with mark levels and basis pattern weights were done using the random-set method [65]. (an implementation of the random-set method is included in the source-code distribution of epicode). Annotations for Ensembl genes were obtained from MSigDB [64] (msigdb.v3.1.entrez.gmt) and re-mapped from Entrez gene ids (EG) onto GENCODE V14 genes [83] using identifier maps (EG to ENSG) from Ensembl (current as of Apr 20th 2013). Association p-values (obtained from the random-set method) were FDR-corrected (BH-method [84]) over the whole 8513 terms in the MSigDB database (which is more stringent), but we reported on associations from different classes of MSigDB gene sets individually, since different types of gene sets have different distributions of association p-values (experimental gene sets are typically more closely correlated than literature-derived).

### Data sources

All raw sequencing data used in the case studies were downloaded from the ENCODE project website as FASTQ files. We included all available histone modification data sets for four cell lines A549, H1ESC, HSMM, and HSMMT, with the exception of H3K36me3 in A549 because of poor reproducibility of this dataset between replicates. Additional Pol2 (ChIP-seq), expression (RNA-seq), and DNase accessibility (Digital Genomic Footprinting (DGF) and DNase-seq) data sets were downloaded for H1ESC. In the case of histone ChIP-seq and DNase accessibility, reads from multiple replicates (BAM files) were combined into a single BAM file using samtools merge. List of all analyzed files in included in in Additional file 12.

### Data processing

We used Bowtie2 [85] with all default settings and indexes for the HG19 genome build ( ftp://ftp.ccb.jhu.edu/pub/data/bowtie2_indexes/hg19.zip) for all alignments. To count exonic RNA-seq reads we used the HTSeq tool [42] with default settings on the GENCODE-provided General Transfer Format (GTF) file. To estimate expresssion levels, read-counts for each gene were normalized by total exon length, averaged over replicate samples, and finally scaled to the 0 to 1 range using the same sigmoid function.

### Supervised machine learning

Predictive modeling (ridge regression, penalized logistic regression) was done using scikit-learn. All model parameters, including penalty type ('l1’ or 'l2’) and regularization strength *C* (1, 2, 5, 10, 50, 100, 500), were trained using 10-fold cross-validation. All cross-validated models used 'l2’ penalty and *C* = 1. Models were evaluated on 20 percent, using scripts included in scikit-learn, on hold-out data which was never used for training or cross-validation.

### Evaluation of initialization methods

Three initialization methods were evaluated NNDSVD, random, and randomized NNDSVD (NNDSVDar). In the random initialization both *W* and *H* matrices are filled with random uniform numbers (0 to 1 range). In the NNDSVDar only zero elements (after NNDSVD) are set to small (close to 0) random numbers. The NNDSVD approach is deterministic and is described in detail in [33]. To evaluate similarity between two *H* matrices we use the Munkres algorithm to establish the minimum cost assignment. To find this minimum it is necessary to pair the most similar rows. Similarity of a pair (cost) is evaluated based on the Euclidean distance. The minimum-cost assignment of basis vector pairs is found using the Munkres algorithm [52]*i.e.* a set of pairs is found that minimizes the global cost. We calculate sparsity using Hoyer’s formula.

## Competing interests

The authors declare that they have no competing interests.

## Authors’ contributions

MC implemented epicode, performed the bioinformatics analyses, and prepared the manuscript. SB supervised research and helped prepare the manuscript. Both authors read and approved the final manuscript.

## Supplementary Material

Additional file 1 Python source-code archive. Click here for file

Additional file 2 **Figure S1 Reconstruction error of NMF runs based on random initializations.** Reconstruction error of 1000 NMF runs plotted as a function of the similarity of the factorization to the reference matrix *H* obtained using NNDSVD. The factor matrices are initialized using random positive numbers. Similarity between two *H* matrices is obtained by calculating the minimum euclidean distance between their basis vectors (see Methods). The approximately linear trend shows that solutions that are most similar to NNDSVD have the smallest reconstruction error. Very few solution with a small reconstruction error are dissimilar to the NNDSVD output. Click here for file

Additional file 3 **Figure S2 Correlation of marks and codes within promoter regions** Heat maps of Spearman’s rank correlation coefficients between marks and NMF basis patterns (codes) in bodies of protein coding genes (marks were mapped in A549 cells). The rows and columns of both heatmaps were sorted using Ward’s clustering and the cosine metric. None of the code-correlations exceeds 0.6, which suggests that all variables should be included in the regression. Click here for file

Additional file 4 **Figure S3 Universality of the** ***H* matrix.** Graphical representation of *H* matrices derived for cell types at various levels of differentiation. Levels of epigenetic marks were quantified at promoters (1 kbp windows which include 900 bp upstream and 100 bp downstream of the TSS) of protein coding GENCODE genes. The "absolute" algorithm was applied for *c* = 6 with all standard settings. H1ESC - H1 human embryonic stem cells, HSMM blast - myoblast cells, HSMM tube - myotube cells. Click here for file

Additional file 5 **Figure S4 Clustering of the** ***W* matrix.** K-means clustering heatmap of the *W* matrix. The K-means algorithm was applied for *k* = 12 to the code weight matrix corresponding to (Figure 2B, *c* = 6) using Euclidean distance and median centroids. Clusters were ordered according to a hierarchical clustering of their medians. Click here for file

Additional file 6 **Figure S5 Epigenetic mark levels at TSS-proximal and TSS-distal Pol2-bound sites.** Box plots of sigmoid-scaled levels of histone modifications at 2 kbp sites centered around a Pol2-peak summit. (up) TSS-proximal sites overlapping a TSS site known to GENCODE. (bottom) TSS-distal sites. Boxes indicate medians and 25th and 75th percentiles. Whiskers extend to 1.5 time the interquartile range (IQR) or roughly the 95th percentile. Click here for file

Additional file 7 Table S1 Classification performance of mark-based and code-based logistic regression in the classification of Pol2-bound sites. Click here for file

Additional file 8 Table S2 Parameters of penalized logistic regression models: supervised classification of Pol2-bound TSS-proximal and TSS-distal sites. Click here for file

Additional file 9 **Figure S6 Coefficients of penalized logistic regressions for the classification of TSS-proximal and TSS-distalPol2-bound sites.** (A) Bar charts of regression coefficients for multivariate (top) and zero-order (bottom) mark based logistic regressions models. (B) Bar charts of regression coefficients for multivariate (top) and zero-order (bottom) code-based logistic regressions models. Click here for file

Additional file 10 **Figure S7 Code-dependent distribution of TSS-proximal and TSS-distal Pol2-bound sites.** Each input site was assigned to the discriminatory epigenetic code for which it had the highest loading. For each code the number of TSS-proximal and TSS-distal Pol2-bound sites is plotted. Click here for file

Additional file 11 Statistical association of epigenetic remodelling patterns and molecular signatures during myoblast differentiation. Click here for file

Additional file 12 List of analyzed datasets. Click here for file
