# Supplementary figures and images for: Combinatorial epigenetic patterns as quantitative predictors of chromatin biology
Source: BMC Genomics. 2014 Jan 28;15:76. doi: 10.1186/1471-2164-15-76 (PMC3922690; doi:10.1186/1471-2164-15-76)

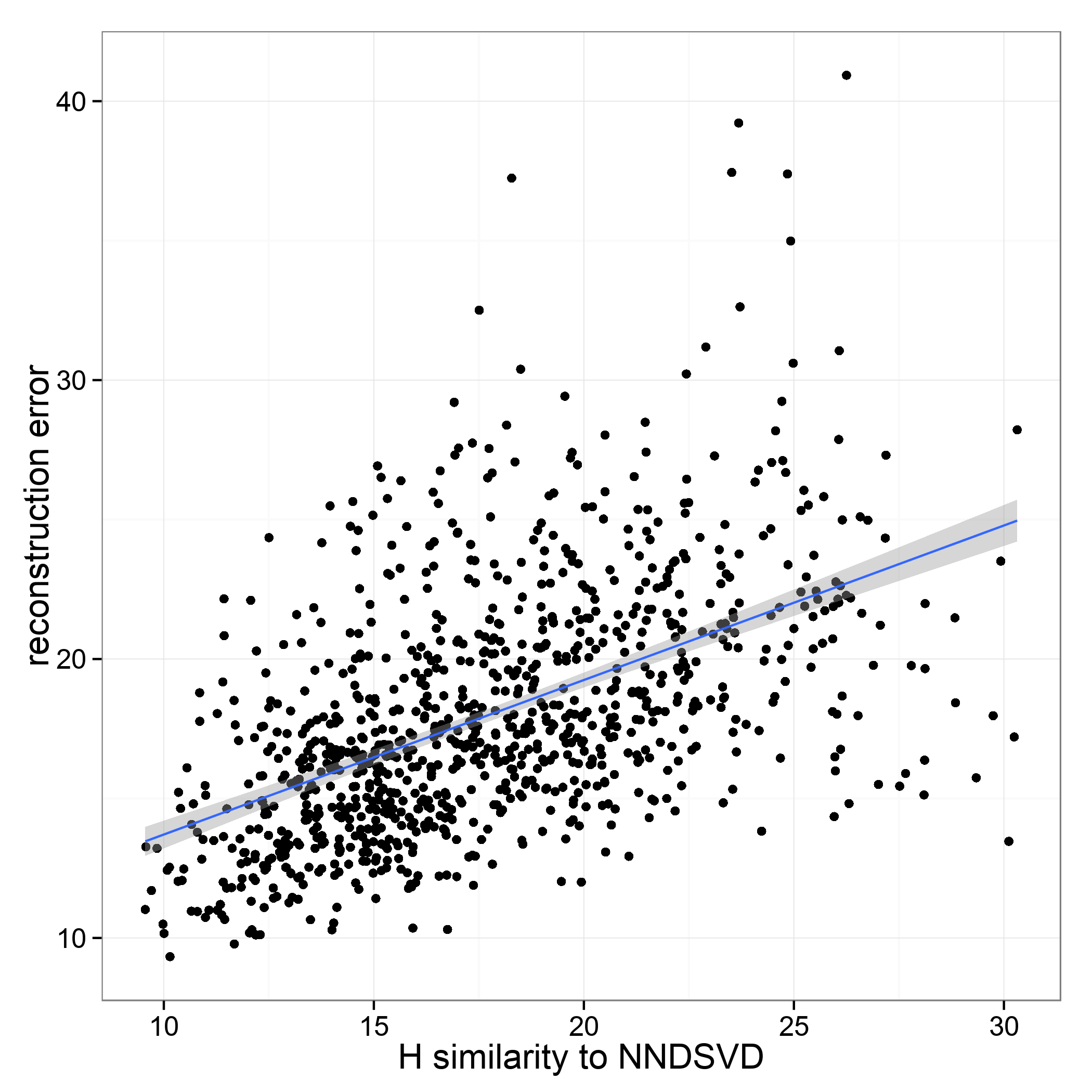

Supplement: Additional file 2 — Figure S1 Reconstruction error of NMF runs based on random initializations. Reconstruction error of 1000 NMF runs plotted as a function of the similarity of the factorization to the reference matrix H obtained using NNDSVD. The factor matrices are initialized using random positive numbers. Similarity between two H matrices is obtained by calculating the minimum euclidean distance between their basis vectors (see Methods). The approximately linear trend shows that solutions that are most similar to NNDSVD have the smallest reconstruction error. Very few solution with a small reconstruction error are dissimilar to the NNDSVD output. [file 1471-2164-15-76-S2.png]

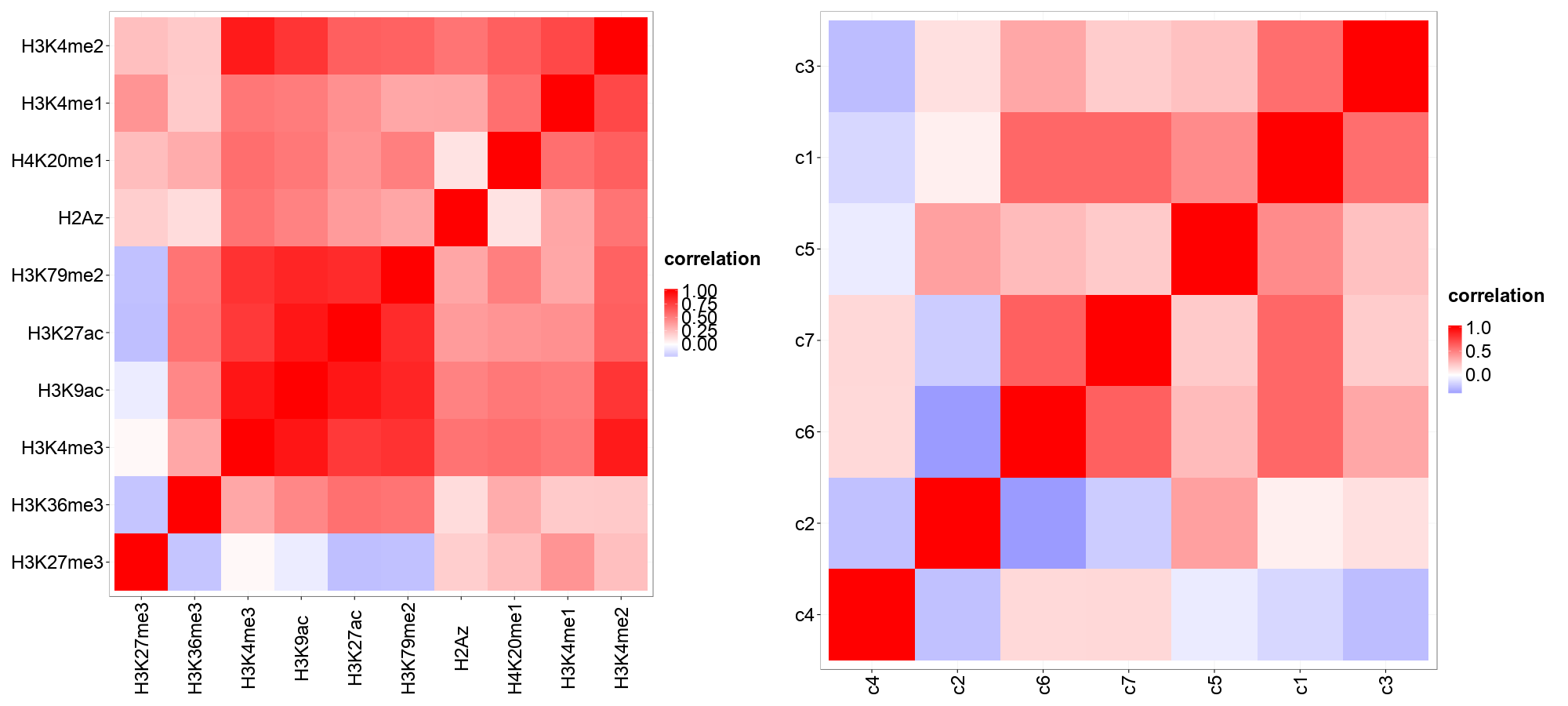

Supplement: Additional file 3 — Figure S2 Correlation of marks and codes within promoter regions Heat maps of Spearman’s rank correlation coefficients between marks and NMF basis patterns (codes) in bodies of protein coding genes (marks were mapped in A549 cells). The rows and columns of both heatmaps were sorted using Ward’s clustering and the cosine metric. None of the code-correlations exceeds 0.6, which suggests that all variables should be included in the regression. [file 1471-2164-15-76-S3.png]

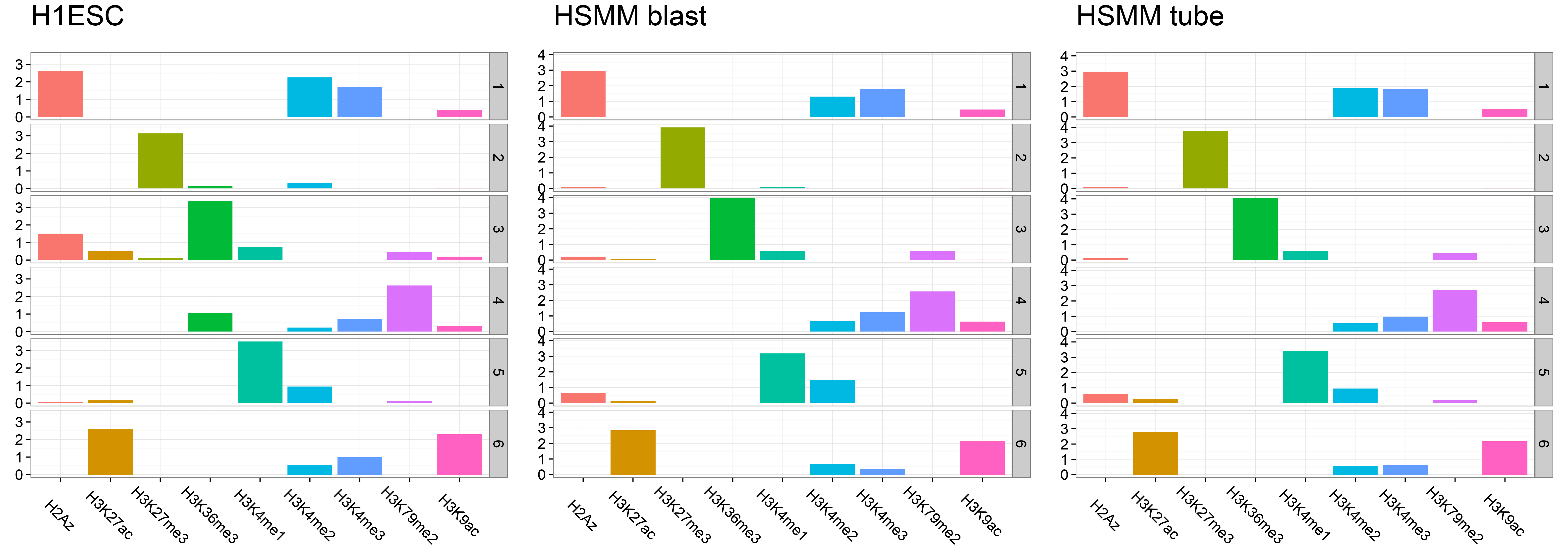

Supplement: Additional file 4 — Figure S3 Universality of the H matrix. Graphical representation of H matrices derived for cell types at various levels of differentiation. Levels of epigenetic marks were quantified at promoters (1 kbp windows which include 900 bp upstream and 100 bp downstream of the TSS) of protein coding GENCODE genes. The "absolute" algorithm was applied for c = 6 with all standard settings. H1ESC - H1 human embryonic stem cells, HSMM blast - myoblast cells, HSMM tube - myotube cells. [file 1471-2164-15-76-S4.png]

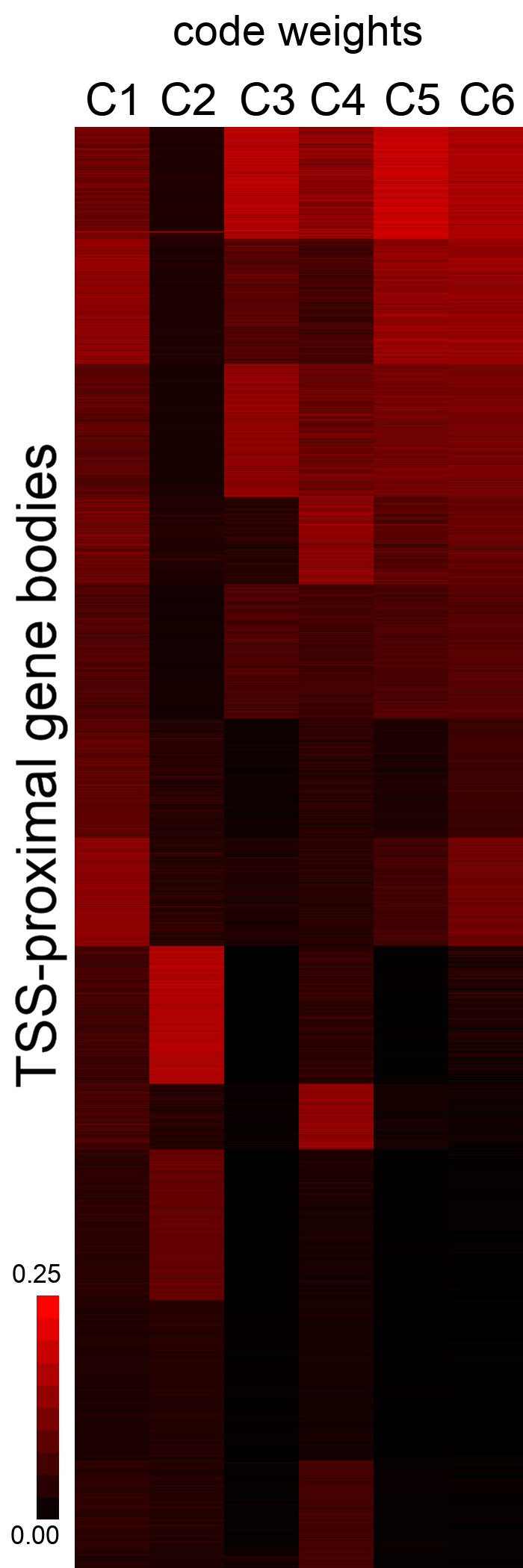

Supplement: Additional file 5 — Figure S4 Clustering of the W matrix. K-means clustering heatmap of the W matrix. The K-means algorithm was applied for k = 12 to the code weight matrix corresponding to (Figure 2B, c = 6) using Euclidean distance and median centroids. Clusters were ordered according to a hierarchical clustering of their medians. [file 1471-2164-15-76-S5.png]

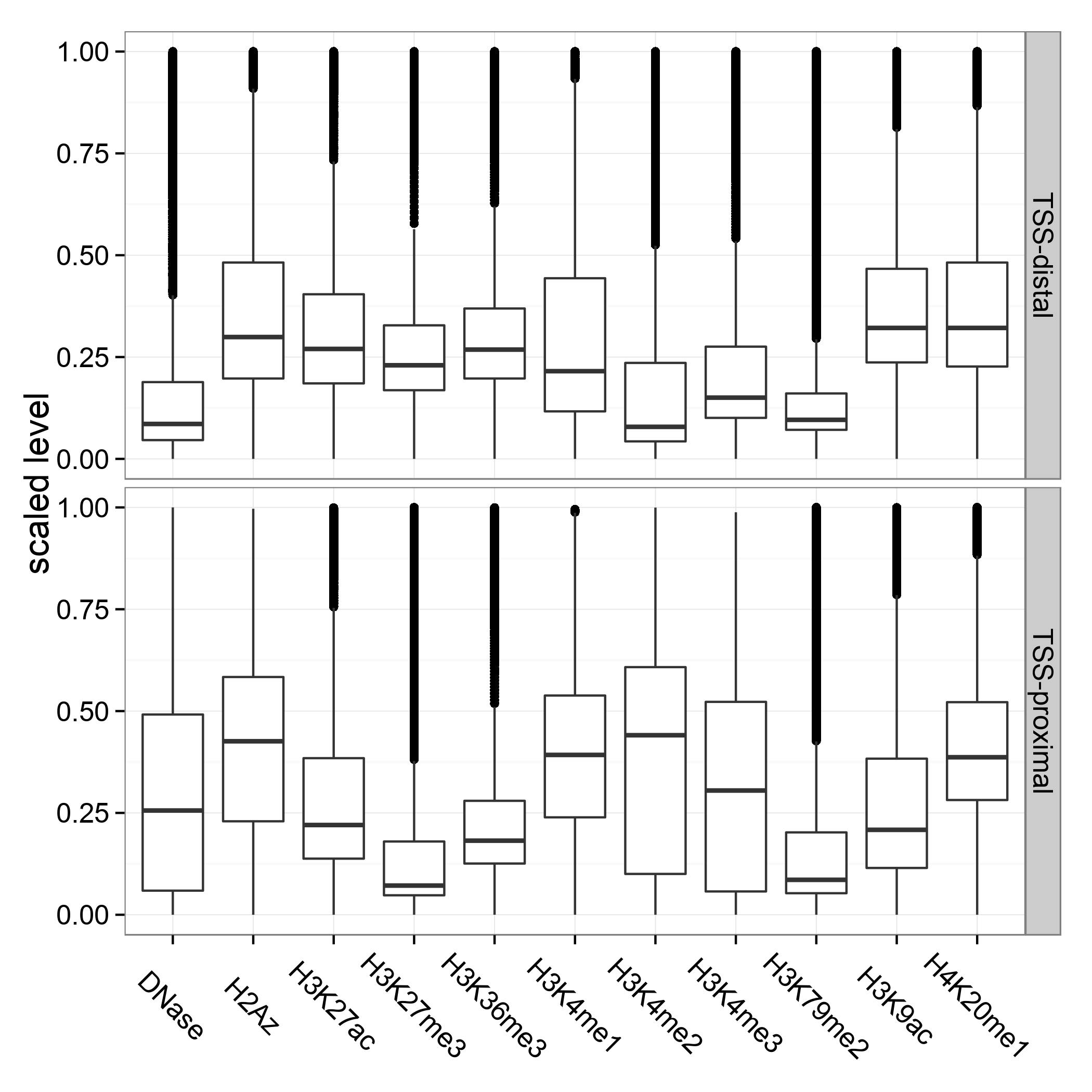

Supplement: Additional file 6 — Figure S5 Epigenetic mark levels at TSS-proximal and TSS-distal Pol2-bound sites. Box plots of sigmoid-scaled levels of histone modifications at 2 kbp sites centered around a Pol2-peak summit. (up) TSS-proximal sites overlapping a TSS site known to GENCODE. (bottom) TSS-distal sites. Boxes indicate medians and 25th and 75th percentiles. Whiskers extend to 1.5 time the interquartile range (IQR) or roughly the 95th percentile. [file 1471-2164-15-76-S6.png]

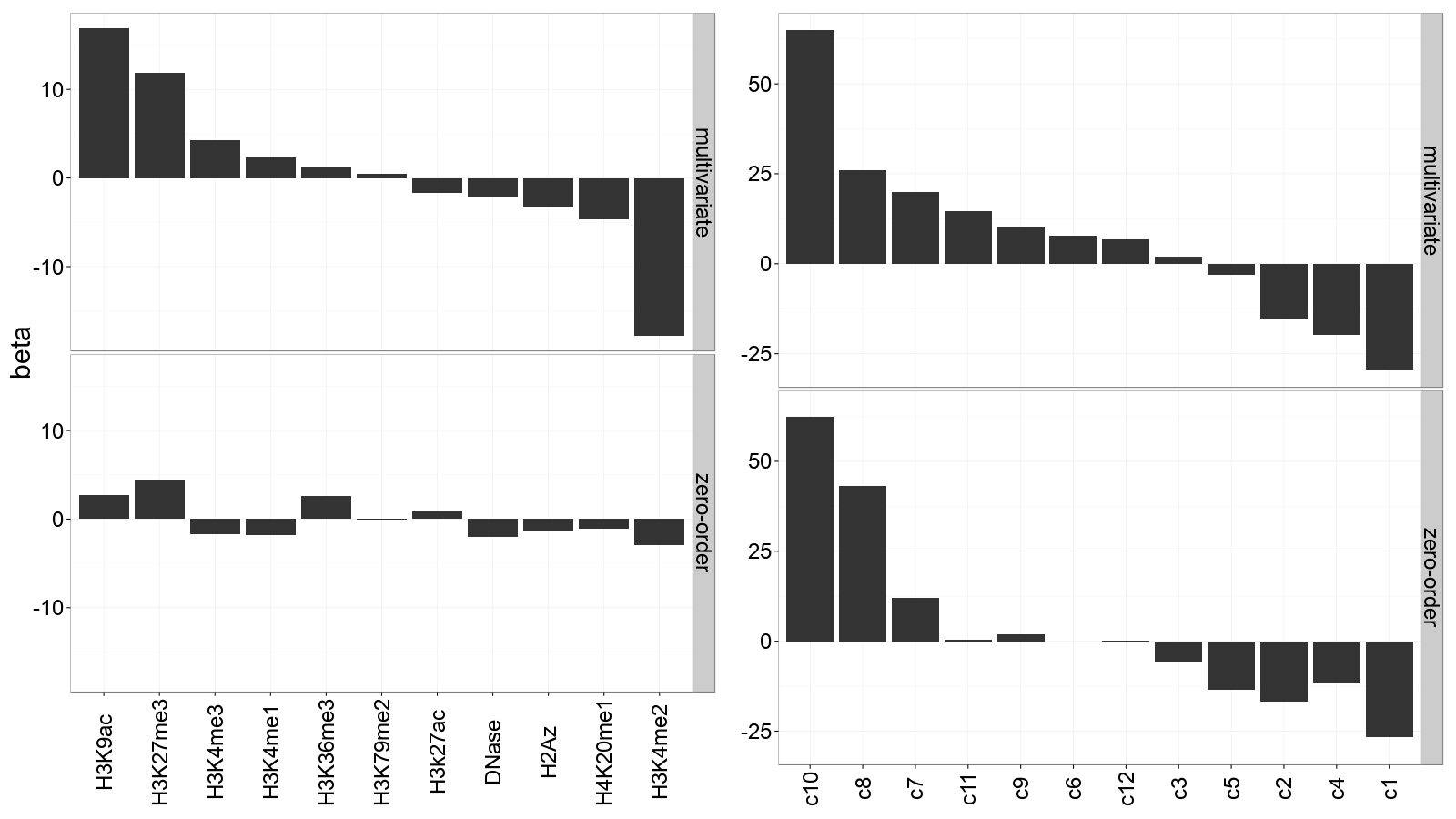

Supplement: Additional file 9 — Figure S6 Coefficients of penalized logistic regressions for the classification of TSS-proximal and TSS-distalPol2-bound sites. (A) Bar charts of regression coefficients for multivariate (top) and zero-order (bottom) mark based logistic regressions models. (B) Bar charts of regression coefficients for multivariate (top) and zero-order (bottom) code-based logistic regressions models. [file 1471-2164-15-76-S9.png]

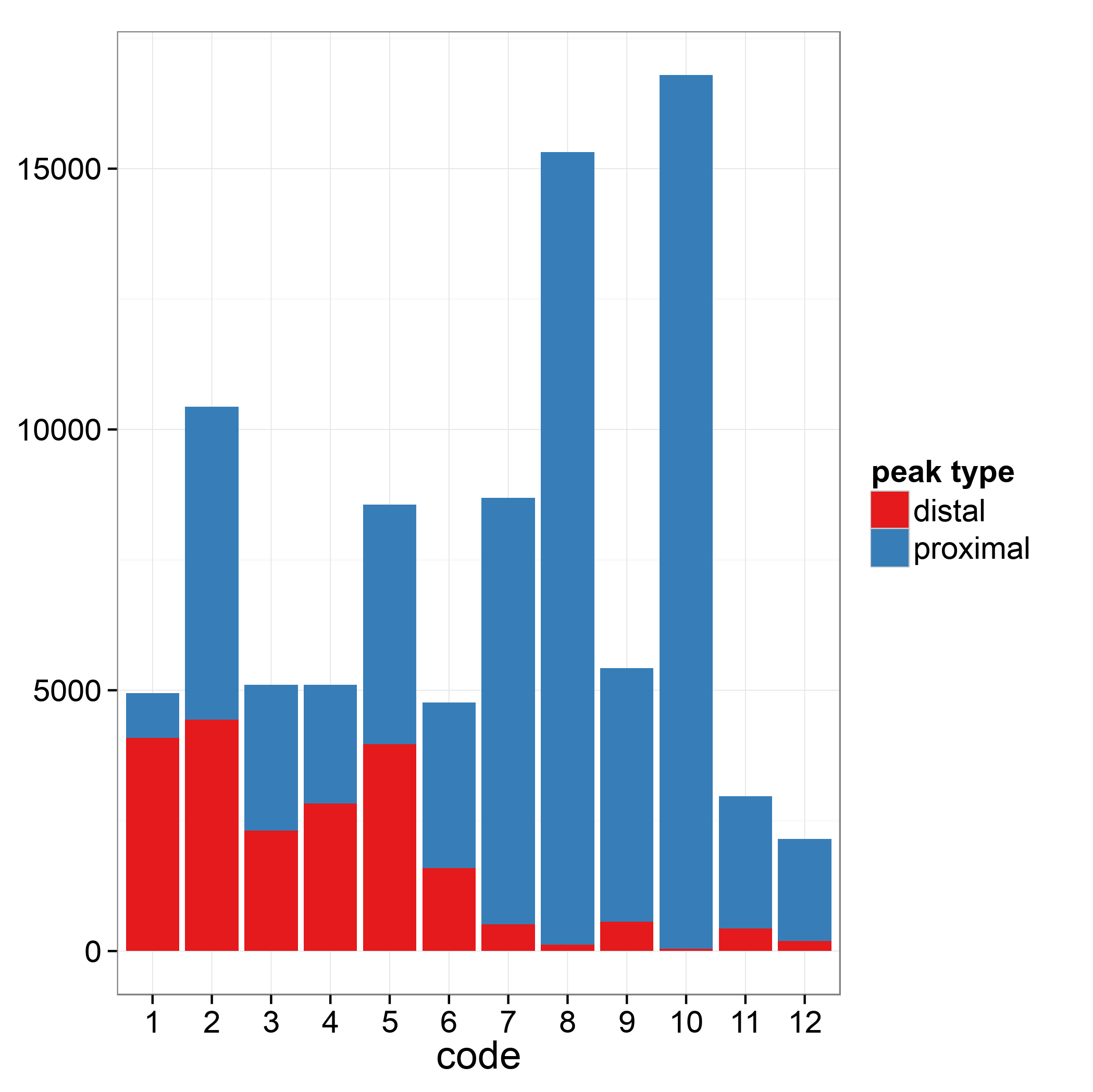

Supplement: Additional file 10 — Figure S7 Code-dependent distribution of TSS-proximal and TSS-distal Pol2-bound sites. Each input site was assigned to the discriminatory epigenetic code for which it had the highest loading. For each code the number of TSS-proximal and TSS-distal Pol2-bound sites is plotted. [file 1471-2164-15-76-S10.png]
