# Supplementary material for: Combinatorial epigenetic patterns as quantitative predictors of chromatin biology
Source: BMC Genomics. 2014 Jan 28;15:76. doi: 10.1186/1471-2164-15-76 (PMC3922690; doi:10.1186/1471-2164-15-76)
Supplement: Additional file 7 — Table S1 Classification performance of mark-based and code-based logistic regression in the classification of Pol2-bound sites. [file 1471-2164-15-76-S7.pdf]

Table S1 - Classification performance of mark-based and code-based logistic regression in the classification of Pol2-bound sites

	mark-based	code-based
MCC	0.82	0.83
AUC	0.96	0.97

AUC – area under the ROC curve; MCC – Matthew's correlation coefficient
