# Supplementary material for: Combinatorial epigenetic patterns as quantitative predictors of chromatin biology
Source: BMC Genomics. 2014 Jan 28;15:76. doi: 10.1186/1471-2164-15-76 (PMC3922690; doi:10.1186/1471-2164-15-76)
Supplement: Additional file 8 — Table S2 Parameters of penalized logistic regression models: supervised classification of Pol2-bound TSS-proximal and TSS-distal sites. [file 1471-2164-15-76-S8.pdf]

Table S2 - Parameters of penalized logistic regression models: supervised classification of Pol2-bound TSS-proximal and TSS-distal sites

variable	beta (multi)	beta (zero-order)	MCC (zero-order)
dnase	-2.07	-2.01	0.30
H2Az	-3.33	-1.40	0.17
H3K27ac	-1.63	0.92	0.18
H3K27me3	11.92	4.37	0.65
H3K36me3	1.20	2.58	0.33
H3K4me1	2.37	-1.82	0.25
H3K4me2	-17.78	-2.93	0.40
H3K4me3	4.29	-1.66	0.27
H3K79me2	0.50	-0.04	0.05
H3K9ac	16.88	2.72	0.40
H4K20me1	-4.71	-1.08	0.13
code 1	-29.63	-26.69	0.50
code 2	-15.39	-16.66	0.34
code 3	2.03	-5.96	0.16
code 4	-19.71	-11.78	0.31
code 5	-3.00	-13.37	0.37
code 6	7.76	-0.09	0.14
code 7	19.97	12.13	0.27
code 8	26.08	43.14	0.55
code 9	10.30	1.84	0.05
code 10	64.93	62.27	0.58
code 11	14.58	0.48	0.07
code 12	6.70	0.07	0.08

Beta coefficients of multivariate and zero-order penalized logistic regression models. MCC – (Matthew's correlation coefficient) is a balanced measure of model performance (higher is better).
